# Energy supply per neuron is constrained by capillary density in the mouse brain

**DOI:** 10.3389/fnint.2022.760887

**Published:** 2022-08-29

**Authors:** aLissa Ventura-Antunes, Suzana Herculano-Houzel

**Affiliations:** ^1^Instituto de Ciências Biomédicas, Federal University of Rio de Janeiro, Rio de Janeiro, Brazil; ^2^Department of Psychology, Vanderbilt University, Nashville, TN, United States; ^3^Department of Neurology, Vanderbilt Medical Center, Nashville, TN, United States; ^4^Department of Biological Sciences, Vanderbilt University, Nashville, TN, United States; ^5^Vanderbilt Brain Institute, Vanderbilt University, Nashville, TN, United States

**Keywords:** metabolism, capillary density, brain vasculature, brain energetics, neuronal density

## Abstract

Neuronal densities vary enormously across sites within a brain. Does the density of the capillary bed vary accompanying the presumably larger energy requirement of sites with more neurons, or with larger neurons, or is energy supply constrained by a mostly homogeneous capillary bed? Here we find evidence for the latter, with a capillary bed that represents typically between 0.7 and 1.5% of the volume of the parenchyma across various sites in the mouse brain, whereas neuronal densities vary by at least 100-fold. As a result, the ratio of capillary cells per neuron decreases uniformly with increasing neuronal density and therefore with smaller average neuronal size across sites. Thus, given the relatively constant capillary density compared to neuronal density in the brain, blood and energy availability per neuron is presumably dependent on how many neurons compete for the limited supply provided by a mostly homogeneous capillary bed. Additionally, we find that local capillary density is not correlated with local synapse densities, although there is a small but significant correlation between lower neuronal density (and therefore larger neuronal size) and more synapses per neuron within the restricted range of 6,500–9,500 across cortical sites. Further, local variations in the glial/neuron ratio are not correlated with local variations in the number of synapses per neuron or local synaptic densities. These findings suggest that it is not that larger neurons, neurons with more synapses, or even sites with more synapses *demand* more energy, but simply that larger neurons (in low density sites) have more energy available per cell and for the totality of its synapses than smaller neurons (in high density sites) due to competition for limited resources supplied by a capillary bed of fairly homogeneous density throughout the brain.

## Introduction

The human brain ranks second amongst organs in absolute energy cost at rest only to the liver (Aschoff et al., 1971; Rolfe and Guy, 1997). Such high cost is commonly attributed to synaptic-mediated neuronal activity (Ames, 2000; Attwell and Laughlin, 2001; Harris et al., 2012), and is entirely met by molecules supplied from the blood and provided to neurons through glial cells whose metabolism is coupled to neuronal activity (Pellerin and Magistretti, 2004; Magistretti, 2006). The high energy cost of the brain, liver, and heart puts them at high risk of damage by diseases and conditions such as aging that compromise metabolism and oxygen supply (Leach and Treacher, 1998). Elucidating what determines the high energetic cost of the brain is thus central to understanding healthy and abnormal brain function.

Brain bioenergetics researchers consider that the high energetic cost of the brain is driven by a steep energetic *requirement* of neurons due to costs directly and indirectly related to synaptic activity and membrane repolarization (Attwell and Laughlin, 2001; Howarth et al., 2010; Harris et al., 2012). However, energetic use can be determined by *demand* only if it is matched dynamically by a non-limiting supply; otherwise, it is constrained by energetic *supply* that limits work. The former is the case of modern cities where the electricity that supplies homes is non-limiting, and demand is free to vary but is always met; the latter is the case in rural homes that already consume most of what little electricity they are supplied with and cannot sustain both an electric shower and air conditioning running simultaneously (Banavar et al., 2002). In biology, this is evident, for example, in hummingbirds, forced into torpor when food is insufficient (Hainsworth et al., 1977).

The brain is known to use a disproportionate amount of the body’s energy budget (Mink et al., 1981), but how much that use is limited by its energetic supply is a key open question. Importantly, there is good reason to suspect that brain energy use at rest is already close to the limits established by the blood supply. First, both local glucose use and blood flow at rest depend linearly on local capillary density in rat (Klein et al., 1986) and monkey (Noda et al., 2002). Task-related variations in local CBF are very small, in the order of 2%, and of at most 8% in primary sensory areas, such that total cerebral blood flow (CBF) remains remarkably constant throughout the day in healthy individuals (Schölvinck et al., 2008; Lin et al., 2010). These small local variations in CBF happen with hardly any capillary recruitment (Kuschinsky and Paulson, 1992; Hudetz, 1997). Additionally, blood flow rates in the brain are already as high at rest as they are in skeletal muscle during exercise (Madsen et al., 1993; Zheng et al., 2014).

Here we examine whether the distribution of capillaries in the brain is consistent with a supply-limiting or a demand-based scenario. To that end, we determine whether capillary density is homogeneous throughout the adult brain regardless of local variations in local neuronal density, which would be expected if capillary density were determined in a system-wide manner by physical scaling limitations (Banavar et al., 2002); or whether capillary density varies coordinately with local neuronal densities, which would be consistent with a plastic, demand-based reorganization of the capillary bed in response to use, whether because sites with more neurons have higher energy use simply because of the larger number of neurons, or because larger neurons (in sites of lower local neuronal densities) require more energy individually (Attwell and Laughlin, 2001). Further, to gain insight on the particular issue of energy availability per neuron, we calculate its proxy, the local endothelial-to-neuronal-cell (E/N) ratio, and examine how it varies locally in the mouse brain depending on local neuronal densities. Finally, we examine whether variations in energy availability per neuron might also be related to local variations in numbers of synapses per neuron, by combining our data with direct counts of synapse densities in identified locations in the mouse brain published recently (Zhu et al., 2018).

## Materials and methods

### Ethics statement

All animal use in this project was approved by the Committee on Ethical Animal Use of the Health Sciences Center (CEUA-CCS), Federal University of Rio de Janeiro (UFRJ), with protocol number 01200.001568/2013-87.

### Experimental design

**FIGURE 1:**
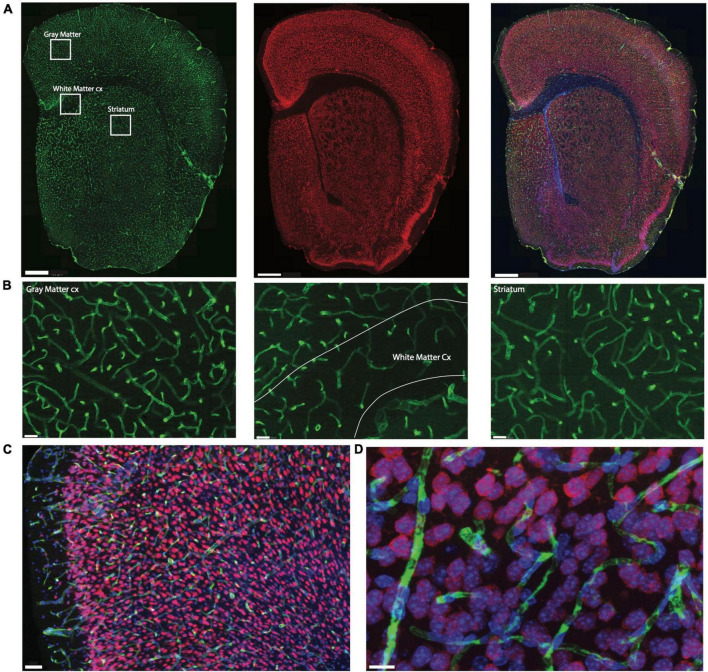
Example of triple labeling for vasculature, neurons, and all cell nuclei. Shown is a coronal section of a mouse brain labeled by systemic injection of FITC-Dextran. **(A)** Coronal section of mouse brain showing microvasculature labeled with injection of FITC-dextran (left, green), neuronal nuclei labeled with NeuN-Cy3 (center, red), and all nuclei labeled with DAPI (right, blue in merged images). Scale bar, 400μm. **(B)** Zoom of insets in **(A)** using the 63× objective with the thinner depth of field illustrating the extent of differences in capillary densities across brain sites. Scale bar, 50 μm. **(C)** Section through cortical gray matter under 20× magnification stained to reveal Collagen IV (green), NeuN (red) and cell nuclei (blue). Scale bar, 50 μm. **(D)** Maximal projection image of 3D stack through cortical gray matter imaged under 63× magnification. Scale bar, 10 μm.

We used structured illumination confocal imaging to allow quantification of cells and microvasculature in individual sections (2D) and stacks (3D) at multiple locations in the brain of five adult mice. In two of those mice, subjected to detailed 3D quantification, brain microvasculature was revealed by injection of a fluorescent tracer (FITC-Dextran, see below) into the caudal vein; in the other three, subjected to much more time-efficient 2D quantification, brain capillaries were revealed by immunohistochemistry against collagen IV, which labels the basal lamina of blood vessels, once we established that measurements of capillary density were indistinguishable between the two labeling methods (see below). Immunohistochemistry against the neuronal marker NeuN (Mullen et al., 1992) was used to reveal neurons; glial cells (astrocytes, oligodendrocytes, and microglia) were identified by exclusion of neurons and capillary-associated cells from the total number of cell nuclei visualized with DAPI (Figure 1).

We quantified capillary area fraction (which is identical to capillary volume fraction) and cell densities in ten easily definable brain structures (cerebral cortical gray and white matter, cerebellar gray and white matter, thalamus, hypothalamus, striatum, hippocampal and cerebellar granular and molecular layers) imaged with structured confocal illumination in a total of 5 mice, comprising 867 stacks analyzed in 3D (two mice, total of 32,270 cells), and 750 ROI acquired and analyzed in 2D (three mice, total of 196,206 cells; Table 1). While image stacks allow detailed visualization of microvasculature in 3D, two-dimensional analysis of image montages allows for larger sites with larger cell populations to be sampled in less time. We thus compared the two approaches in this study to determine whether 2D analysis of the distribution of capillaries and neurons is a viable and more efficient alternative to 3D quantification.

**TABLE 1:** Numbers of cells, stacks and ROIs analyzed in each of ten structures of interest in the mouse brain.

Structure	Mouse 01	Mouse 02	Mouse 03	Mouse 04	Mouse 05
CerCx, GM	32 stacks 2,516 cells	54 stacks 3,495 cells	76 ROI 63,022 cells	57 ROI 17,613 cells	34 ROI 12,053 cells
CerCx, WM	60 stacks 1,497 cells	69 stacks 2,531 cells	59 ROI 2,651 cells	19 ROI 1,636 cells	27 ROI 3,931 cells
Thalamus	83 stacks 4,349 cells	42 stacks 1,997 cells	21 ROI 9,096 cells	15 ROI 5,442 cells	16 ROI 3,634 cells
Hypothalamus	45 stacks 2,816 cells	34 stacks 1,861 cells	13 ROI 5,358 cells	12 ROI 2,260 cells	11 ROI 2,125 cells
Striatum	59 stacks 3,283 cells	43 stacks 2,693 cells	29 ROI 15,539 cells	16 ROI 6,284 cells	18 ROI 6,467 cells
Hippocampus, ML	40 stacks 137 cells	73 stacks 202 cells	9 ROI 136 cells	9 ROI 88 cells	8 ROI 118 cells
Hippocampus, GL	25 stacks 470 cells	21 stacks 368 cells	13 ROI 726 cells	13 ROI 310 cells	9 ROI 609 cells
Cerebellum, GL	37 stacks 2,105 cells	23 stacks 1,167 cells	43 ROI 16,850 cells	22 ROI 4,084 cells	34 ROI 5,892 cells
Cerebellum, ML	41 stacks 361 cells	26 stacks 179 cells	42 ROI 3,982 cells	23 ROI 1,313 cells	35 ROI 1,632 cells
Cerebellum, WM	32 stacks 138 cells	28 stacks 105 cells	29 ROI 1,746 cells	15 ROI 778 cells	23 ROI 831 cells
Total	454 stacks 17,672 cells	413 stacks 14,598 cells	334 ROI 119,106 cells	201 ROI 39,808 cells	215 ROI 37,292 cells

CerCx, cerebral cortex; GM, gray matter; WM, white matter; GL, granular cell layer; ML, molecular cell layer.

**FIGURE 2:**
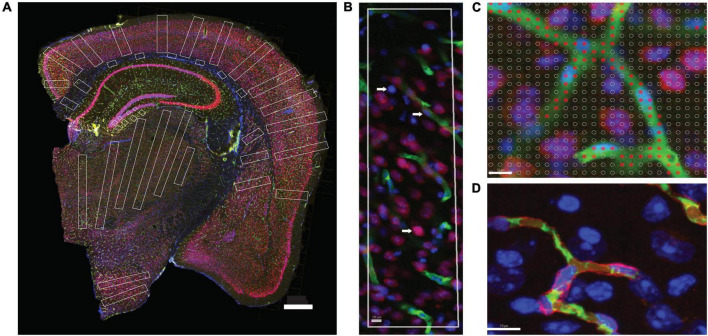
Two-dimensional image analysis. **(A)** Areas sampled for 2D quantification are shown in white across structures in a coronal section of the mouse brain. Scale bar, 500 μm. **(B)** ROI throughout the cortical gray matter, from pial surface to white matter interface, showing microvasculature labeled with anti-collagen IV (green), neurons labeled with anti-NeuN (red) and DAPI to reveal all cell nuclei (blue). Cell nuclei appearing inside green-labeled blood vessels are considered capillary-associated nuclei (and therefore, “endothelial cells”); cell nuclei double-labeled in red are considered neuronal cell nuclei; all remaining nuclei are identified by exclusion as glial cell nuclei. **(C)** A Cavalieri estimator grid of 4 μm spacing (shown) was applied to images acquired under 20× magnification, and a 2 μm grid was applied to images acquired under 63× magnification. The points marked in red correspond to the area of the image occupied by capillaries. Scale bar, 10 μm **(D)**. Superposition of FITC-dextran (green, lumen) and collagen IV (red, basal lamina) labeling of capillaries. Single image acquired under 63× magnification. Scale bar, 10 μm.

From all DAPI-labeled cell nuclei in each stack or 2D ROI, we identified all NeuN-positive cell nuclei as neurons; all nuclei directly associated with collagen IV-labeled or FITC-dextran filled capillaries as endothelial and associated cells (which includes eventual pericytes; heretofore, we refer to all capillary-associated cells as “endothelial cells”), and by elimination, all remaining nuclei were deemed glial cells (Figure 2). Cell densities were calculated as cell nuclei per mm^3^ or mm^2^, depending on the stack volume or ROI area analyzed to include capillaries only. All results are shown side by side in 2D and 3D analyses.

### Experimental animals

Five male Swiss mice aged 2.5 months were analyzed. Two mice received an injection of 200 mg/kg of fluorescein-isothiocyanate-dextran (FITC-dextran 70 kDa) in the caudal vein, to label the lumen of all blood vessels (Figure 1). One hour after the injection, the half-life required for distribution in the lumen of blood vessels (Wang et al., 2016), the animals were deeply anesthetized with Xylazine (15 mg/Kg) and Ketamine (100 mg/Kg), decapitated, and their brain was quickly removed and fixed by immersion in 4% of paraformaldehyde in phosphate buffer (PB) for 1 h followed by 30% sucrose in 0.1 M PB for cryoprotection. Using a Leica microtome, the cerebellum was cut into a series of 40 μm thick sagittal sections, and the remaining tissue was cut into a series of 40 μm coronal sections. All sections were stored at –20^°^C in antifreeze solution (30% polyethylene glycol and 30% glycerol in 0.1 M PB) until further use. Three other mice were similarly deeply anesthetized with Xylazine and Ketamine and perfused through the heart with 0.9% saline followed by 4% paraformaldehyde in PB. The brain was removed and post-fixed for 1 h by immersion in 4% of paraformaldehyde in PB, then sectioned as above.

### Immunofluorescence

One in every six sections of each brain was subjected to immunohistochemistry and counterstaining with DAPI. Anti-NeuN antibody was used to reveal neurons; additionally, those sections from the three animals not injected with FITC-Dextran were subjected to immunohistochemistry against collagen IV to reveal cell nuclei intimately associated with capillaries (Figure 1).

Each section was washed in PB for 5 min, heated for 1 h at 70^°^C in a 0.1 M solution of boric acid, pH 9.0, then incubated for 1 hour in PB containing 3% normal goat serum (Sigma-Aldrich, LG9023) and 2% bovine serum albumin (Sigma-Aldrich- A2058). Sections from animals not injected with FITC-Dextran were next incubated under stirring for 48 h at 4^°^C in PB containing rabbit polyclonal anti-collagen IV antibody (Abcam, Ab6586) at a 1:500 dilution. Each section was then washed three times in PB for 5 min each and incubated for 2 h at 4^°^C in a 1:500 dilution of Alexa Fluor 488 goat anti-rabbit secondary antibody (Abcam, Ab150077). From this step on, all brain sections were treated similarly: washed three times in PB and incubated for 24 h at 4^°^C under continuous stirring with rabbit primary polyclonal antibody against Cy3-conjugated NeuN (Millipore, ABN78C3) diluted 1:1,000 in PB. All sections were then labeled with DAPI (4′,6-Diamidino-2-Phenylindole Dilactate, from stock solution at 20 mg/l) to provide counter-staining for identification of brain structures and allow visualization of all cell nuclei in each section. The sections were then mounted on glass slides and coated with Vectashield (Vector Labs, Cat. number H-1400) for viewing under fluorescence microscopy.

### Structures analyzed

The ten structures of interest targeted for analysis [gray matter (GM) and white matter (WM)] of the cerebral cortex; striatum; thalamus; hypothalamus; granular and molecular layers of the hippocampal dentate gyrus; WM and granular and molecular layers of the cerebellum) were defined according to the Mouse Brain Atlas (Franklin and Paxinos, 1997). To capture values that are representative of the different regions of the cerebral cortex, irrespective of layer-specific variations in capillary density (Tsai et al., 2009), all ROIs placed in the GM of the cerebral cortex were long rectangles that spanned all layers, from the pial surface to the GM-WM interface (Figure 2).

### Microscopy

Two Zeiss AxioImager M2 microscopes equipped with an Apotome 2 for confocal imaging under structured illumination (Carl Zeiss, Jena, Germany) and driven by StereoInvestigator software (Microbrightfield Bioscience, Williston, VT) were used for all image acquisition.

We first delineated the boundaries of the ten brain structures of interest in each section. For each structure, image stacks for 3D analysis were acquired using systematic random sampling (SRS) in Stereo Investigator under magnification with a 63× objective (Plan-ApoChromat), which gives a depth of field of 0.5 μm with oil, with an optical plane of 0.5 μm and step size of 0.5 μm. Stacks were 1,388 × 1,040 μm wide × 30 μm thick and typically contained 60 images each, spanning most of the full mounted thickness of the section. Within each stack, 3D counting probes placed were variable in size according to the structure (Table 2) and were placed to exclude the sectioned surfaces and zones outside the structure of interest. The gray matter of the cerebellar cortex was imaged as a whole and analyzed separately into molecular and granular layers. For 2D analysis, image composites of the entire brain sections were acquired under 20× magnification (EC Plan-NeoFluar/420350-9900), which gives a thicker depth of field of ca. 4 μm, and counting probes consisted of rectangular regions of interest (ROIs) placed to cover large areas of the target structures in each brain section (see Figure 2). In the cerebral cortex, rectangular probes spanned the entire gray matter from pia to the WM border, and 5–15 probes of ca. 800 μm width were placed at regular intervals along each section. For all other structures, 2–8 rectangular probes were placed in each section, seeking to maximize the sampled surface of each structure (Figure 2A).

**TABLE 2:** Size of 3D counting probes per structure.

CerCx GM	X 127 y 96 z 20 μm
CerCx WM	x 80 y 75 z 20 μm
Thalamus	x 100 y 95 z 20 μm
Hypothalamus	x 100 y 95 z 20 μm
Striatum	x 100 y 95 z 20 μm
Hippocampus ML	x 40 y 40 z 20 μm
Hippocampus GL	x 30 y 30 z 20 μm
Cerebellum GL	x 40 y 40 z 20 μm
Cerebellum ML	x 40 y 40 z 20 μm
Cerebellum WM	x 40 y 40 z 20 μm

CerCx, cerebral cortex; GM, gray matter; WM, white matter; GL, granular cell layer; ML, molecular cell layer.

Although highly time-consuming, detailed analysis of 3D images was performed first to rule out bias in data acquisition due to any preferential organization of capillaries relative to the plane of section, given the three-dimensional nature of the distribution of the capillary network. To ensure feasibility of future analyses of a large number of species, and to allow for a much larger sample size, we compared the results of the 3D analysis of small image stacks with the much faster and more practical analysis of 2D image composites, which permitted the analysis of much larger ROIs and numbers of cells in half as much time. Those analyses had to be performed in separate animals due to bleaching of the immunofluorescence during the acquisition of image stacks. Due to software limitations that precluded acquiring large composites at 63×, 2D image composites had to be acquired at 20×, which leads to inflated estimates of area (or volume) fraction occupied by capillaries due to the projection of oblique vessels given the larger thickness of the optical sections at 20× (ca. 4 μm) compared to 63× (ca. 0.5 μm). For the same reason, estimates of local cell densities will also differ between the two methods. However, we hypothesize that all relationships across variables should remain unaffected by the method of image acquisition. All results are thus presented side by side for 3D and 2D analyses. Data points overlap across the five animals (shown in different symbols in all figures), and are thus analyzed jointly.

### 3D analysis

Stacks in the targeted brain structures of two FITC-injected mice were acquired using systematic random sampling (SRS) of each structure in each of 1 of 6 sections. From each brain, 13 and 18 sagittal sections through the cerebellum and 24 and 23 coronal sections through the remaining brain structures, including the cerebrum, were analyzed. A total of 867 stacks were analyzed across the 10 brain structures in two mice (Table 1, mouse 01 and mouse 02).

### 2D analysis

From each of three animals, 21, 12, and 17 sections through the cerebellum and 21, 20, and 14 sections through the remaining brain structures were analyzed. A total of 750 ROIs were imaged and analyzed across the ten brain structures (Table 1, mice 03–05).

### Image analysis

In each stack and ROI analyzed, we manually identified each DAPI-stained cell nucleus in the structure site as neuronal (when it expressed NeuN immunoreactivity), endothelial or belonging to other capillary-associated cells (when the cell was intimately associated with FITC-labeled capillary lumen or collagen IV-stained basal lamina, which includes pericytes; all of these are referred to as “endothelial cells”), or glial (by exclusion; Figure 2B).

Microvascular area fraction (hereafter termed “capillary area fraction” for simplicity) was estimated in image stacks and 2D ROIs using the Cavalieri estimator in StereoInvestigator using a grid of points separated by 2 μm in 3D images, and 4 μm in 2D images (Figure 2C). Capillary *area* and *volume* fraction are interchangeable (Gundersen et al., 1999) and refer to the fraction of tissue formed by endothelial cells and the lumen of the capillaries that they form. In order to restrict analysis to capillaries, wherever stacks included vessels larger than the typical capillary diameter, the counting probe was reduced to avoid it, or else the stack was discarded. In 2D, ROIs were placed to avoid large vessels.

Labeling capillaries with FITC-dextran or immunohistochemistry to collagen IV is expected to lead to slightly different measurements of capillary density expressed as area (or volume) fraction, because while the former labels the lumen of blood vessels, the latter labels the basal lamina of the cells that form the walls of the vessels. However, estimates of numbers of capillary-associated cells per site should remain unaffected. To evaluate how much the difference in measured capillary volume fraction depending on staining method would impact our results, one additional 1-in-6 series of brain sections of mouse #01, which received the injection of FITC-Dextran, were double-labeled for collagen IV using the same primary antibody as before, but a different secondary antibody, conjugated to Alexa Fluor 546 (1:500/#A11010 - Life), resulting in coincident but non-overlapping double labeling of lumen and basal lamina of the microvasculature and the associated DAPI-stained cell nuclei (Figure 2D). Quantification of the area fraction covered by capillaries in 2D composites acquired at 20× showed a detectable but not statistically significant difference between the microvascular fraction estimated with collagen IV (7.4 ± 0.3%) compared to FITC-Dextran (6.6 ± 0.4%). We thus combined the data on microvascular (capillary) fraction across all animals.

### Correlations with synaptic density

In order to determine whether local densities of synapses correlate with local densities of neurons and capillaries and to establish how variable are the ratios of synapses per neuron and number of synapses supplied per capillary cell, we proceeded with the quantification of four sections from the brain of mouse #4 that matched the respective levels of the four coronal sections through the cerebral cortex of one individual mouse in the synaptome study of Zhu et al. (2018). We used the Mouse Brain Atlas (Franklin and Paxinos, 1997) to place large ROIs in the same structures. 2D composite images acquired under 20× magnification were analyzed to determine the density of cells and vascular fraction of neocortex in our images for comparison with the total number of synapses labeled with either PSD95 or SAP (Zhu et al., 2018).

### Statistical analysis

All analyses were performed in JMP14 PRO, using non-parametric Spearman correlation coefficients to test the correlation between variables, and regression to mathematical functions to determine the type of relationship between variables.

## Results

**FIGURE 3:**
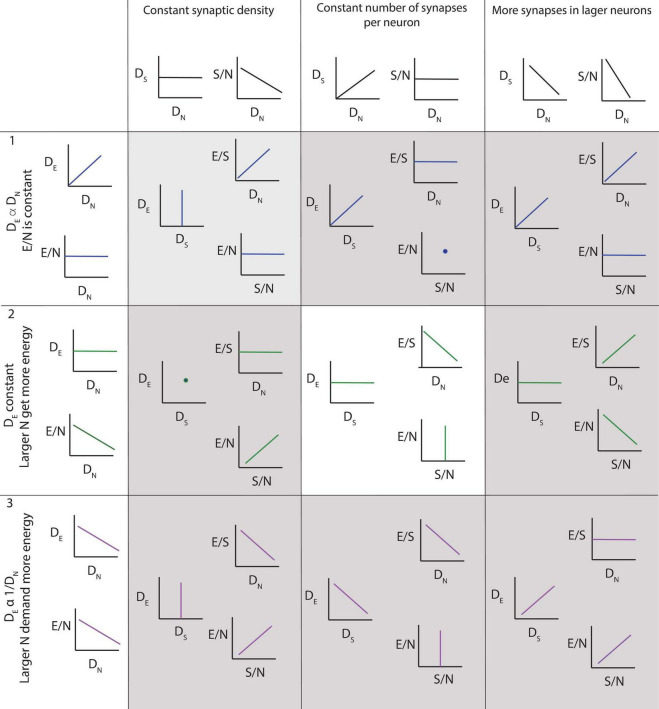
Three of several possible scenarios of relationships among local density of capillary cells (D_E_), local density of neurons (D_*N*_), energy available per neuron (E/N, estimated as D_E_/D_N_), local density of synapses (D_S_), average number of synapses per neuron (S/N) and average energy supply per synapse (E/S). **Scenario 1 (top row):** neurons demand a certain fixed supply of energy, and the capillary bed adjusts to supply it at a steady-state, such that energy supply per neuron is constant across sites. D_E_ is expected to be proportional to D_N_, and energy available per neuron (E/N) is constant. Scenario 2 (second row): capillary supply to brain tissue is constant and neurons compete for energy available. In this case, D_E_ is expected to be relatively constant regardless of local D_N_; as a result, E/N is larger in sites of lower neuronal densities, where fewer neurons compete for a limited supply of energy. This is the scenario supported by our findings, but incompatible with current models. Scenario 3 (third row): larger neurons demand more energy, and the capillary bed adjusts to supply it, such that energy supply per neuron is larger where neurons are also larger, and thus occur at lower D_N_. This scenario is compatible with current models, but is not supported by our present findings. Columns indicate, for each scenario of capillary and neuronal distribution, the expected findings regarding variations across the variables measurable in this study depending on whether (left column) local synaptic densities are mostly constant across sites, such that there are more synapses per neuron where neurons are larger (and D_N_ lower); (center column) the number of synapses per neuron is mostly constant, such that there are more synapses where there also are more neurons; and (right column) larger neurons have many more synapses than smaller neurons, such that local synaptic densities are larger where neurons are larger (and D_N_ lower). Our findings support a relatively constant density of capillaries with fairly constant densities of synapses per neuron, such that energy supply both per neuron and per synapse, and thus energy availability per neuron and per synapse, decreases the higher the local neuronal density, that is, the smaller the average local volume of individual neuronal cells. This is the scenario highlighted in the center panel.

To guide the interpretation of our findings, the three main possible scenarios are depicted in Figure 3. The top row illustrates the results expected if the steady-state capillary density in the adult brain reflected local variations in neuronal density, and the average neuron was supplied with similar amounts of energy regardless of its size or location. In this case, local capillary density should be directly proportional to local neuronal density, and energy availability per neuron, indicated by the ratio of endothelial-associated (or simply “endothelial”) cells per neuron, should remain constant across brain sites. On the other hand, if larger neurons are supplied with more energy, then sites with larger neurons and thus lower neuronal densities should have higher E/N (Figure 3, rows 2 and 3). However, such finding of more energy availability where neurons are larger could occur in two different scenarios. In the first, higher E/N would happen due to reorganization of the capillary bed reflecting neuronal *demand*, in which case larger capillary densities would be expected in those sites with larger neurons (and therefore lower neuronal densities; row 3). In the second, higher E/N in those sites with larger neurons would be simply imposed by a lack of variation in capillary density across brain sites (row 2). The three particular cases in which numbers of synapses are constant per volume (left), constant per neuron (center) or larger in bigger neurons (right) are depicted separately for each scenario and illustrate the ensuing expected variation in energy availability per synapse as well as how energy availability per neuron would vary together with energy availability per synapse or not.

Across the ten brain structures examined, we find that the capillary area fraction is very small, amounting to no more than 3% of the tissue in any structure in 3D image stacks. While individual measurements of local capillary fraction vary 10x-fold across all brain sites, the average capillary fraction per structure varies only about twofold across structures, with 95% of measurements ranging around 1.29 ± 1.39% (Table 3). The average capillary fraction is highest in the GM of the cerebral cortex (2.22 ± 0.11%), and lowest in the granular layer of the dentate gyrus and subcortical WM (0.70 ± 0.07%; Table 3). In 2D images, we found similarly small variation in capillary fraction, as well as lower capillary fractions in WM than in GM, despite the artificially higher nominal capillary fractions measured with this method due to the larger optical thickness of the images (Table 4). The capillary fractions we report in 3D analyses are similar to vascular fractions between 2 and 6% reported in the mouse cerebral cortical gray matter (Tsai et al., 2009) and between 2.1 and 3.1% in the cat, macaque and human GM cortex (Pawlik et al., 1981; Borowsky and Collins, 1989; Boero et al., 1999).

**TABLE 3:** Fractional cellular composition of different structures of the mouse brain, quantified in 3D.

3D	% capillary fraction	% endothelial cells	% neurons	% glial cells	Glial cells% of non-neuronal cells
CerCx, GM	2.22 ± 0.11%	13.90 ± 0.79%	57.86 ± 1.53%	28.22 ± 1.23%	66.64 ± 1.49%
CerCx, WM	0.75 ± 0.03%	7.62 ± 0.60%	5.75 ± 1.15%	82.61 ± 1.41%	91.37 ± 0.76%
Striatum	1.43 ± 0.06%	7.94 ± 0.34%	62.47 ± 1.10%	29.57 ± 1.10%	77.58 ± 0.99%
Thalamus	1.35 ± 0.05%	10.74 ± 0.52%	51.24 ± 1.31%	38.01 ± 1.2%	77.31 ± 1.00%
Hypothalamus	1.34 ± 0.06%	10.14 ± 0.69%	60.62 ± 1.61%	29.23 ± 1.36%	73.34 ± 1.64%
DG, GL	0.70 ± 0.07%	1.46 ± 0.47%	94.96 ± 1.08%	3.57 ± 0.92%	67.46 ± 9.30%
DG, ML	1.10 ± 0.06%	29.18 ± 3.21%	10.33 ± 3.29%	60.48 ± 3.29%	68.23 ± 3.35%
Cb, GL	1.56 ± 0.06%	2.58 ± 0.33%	95.44 ± 0.46%	1.96 ± 0.33%	38.86 ± 5.06%
Cb, ML	1.69 ± 0.09%	13.92 ± 3.76%	59.77 ± 3.90%	26.30 ± 3.76%	54.35 ± 4.93%
Cb, WM	1.25 ± 0.06%	14.14 ± 2.68%	0.73 ± 0.52%	85.12 ± 2.76%	85.64 ± 2.69%

Values correspond to the average across sites in each structure of two mice (#1 and #2). CerCx, cerebral cortex; GM, gray matter; WM, white matter; GL, granular cell layer; ML, molecular cell layer; Cb, cerebellum.

**TABLE 4:** Fractional cellular composition of different structures of the mouse brain, quantified in 2D.

2D	% capillary fraction*	% endothelial cells	% neurons	% glial cells	Glial cells% of non-neuronal cells
CerCx, GM	8.65 ± 0.21%	14.37 ± 0.23%	60.86 ± 0.39%	24.75 ± 0.36%	63.06 ± 0.53%
CerCx, WM	5.35 ± 0.27%	10.63 ± 0.55%	2.99 ± 0.93%	86.37 ± 1.18%	88.55 ± 0.75%
Striatum	7.74 ± 0.25%	11.88 ± 0.37%	63.59 ± 0.83%	24.51 ± 0.71%	67.14 ± 0.88%
Thalamus	7.58 ± 0.71%	14.53 ± 0.71%	45.88 ± 1.89%	39.57 ± 1.90%	72.19 ± 1.30%
Hypothalamus	7.28 ± 0.39%	12.38 ± 0.63%	57.13 ± 1.60%	30.47 ± 1.34%	70.77 ± 1.40%
DG, GL	6.17 ± 0.06%	4.55 ± 0.72%	90.89 ± 1.46%	4.55 ± 0.89%	46.32 ± 5.72%
DG, ML	10.91 ± 0.91%	30.67 ± 1.70%	13.17 ± 2.34%	56.15 ± 2.34%	64.43 ± 1.93%
Cb, GL	6.76 ± 0.26%	3.02 ± 0.17%	91.00 ± 0.47%	5.97 ± 0.38%	63.22 ± 1.83%
Cb, ML	7.20 ± 0.25%	18.77 ± 0.62%	47.02 ± 1.57%	34.20 ± 1.48%	62.47 ± 1.48%
Cb, WM	4.16 ± 0.22%	13.50 ± 0.77%	2.34 ± 0.56%	84.14 ± 0.95%	86.13 ± 0.79%

Values correspond to the average across sites in each brain structure of three mice (#3, #4 and #5). *Note that the capillary fraction appears higher here than when measured in 3D due to the deeper focal thickness under the lower magnification used for 2D image acquisition, which artificially inflates the relative capillary fraction. Other values are similar between 2D and 3D estimates.

Interestingly, capillary-associated (“endothelial” cells) typically constitute between 7 and 14% of all cells forming the brain structures examined (Tables 3, 4), a much larger percentage than could be expected from the average vascular fraction of 0.7–2.2% of the 3D volume that they occupy. This discrepancy indicates that endothelial cells are, on average, much smaller than neurons and glial cells in the tissue. Still, glial cells are consistently the majority (60–70%) of non-neuronal cells in all structures (Tables 3, 4). As expected from our previous studies (Herculano-Houzel et al., 2013, 2014), the percentage of cells that are neurons is highly variable across structures and sites within a structure; neurons are the vast majority of all cells in the granular layers of the dentate gyrus and cerebellum, a smaller percentage of all cells in other GM structures, and rare (but present) within the subcortical WM of both cerebral and cerebellar cortices (Tables 3, 4).

**FIGURE 4:**
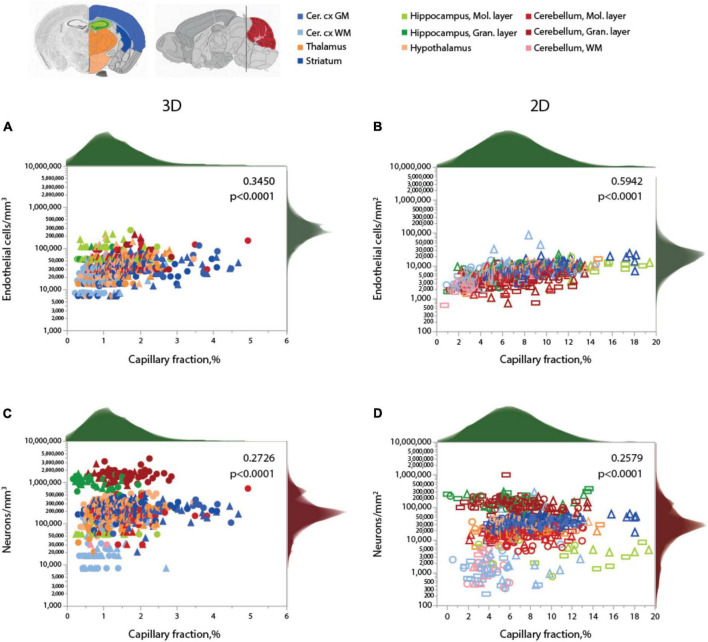
Local capillary fraction correlates with local endothelial cell density, but not with neuronal cell density. **(A,B)** Local density of endothelial cells correlates with local vascular fraction across all sites and structures analyzed in 3D (**A**; two animals, shown in circles or triangles, left) and in 2D **(B)**; three animals, shown in circles, triangles, or rectangles, right) with the Spearman correlation coefficients and *p*-values across all animals and sites indicated in each graph. **(C,D)** In contrast, local neuronal density does not correlate significantly with local vascular fraction across brain sites and structures in 3D **(C)** or in 2D **(D)**. Histograms above and to the right reflect the distribution of all data points in each axis. Graphs in **(A,C)** and **(B,D)** are shown with similar X and Y scales for comparison. Correlation coefficients and power exponents for each structure are given in Table 6.

As expected for two different measurements of the same feature (capillary density), local endothelial cell density correlates well with local capillary area or volume fraction within and across the different brain structures, as well as across animals, especially in the 2D sample, which included a much larger number of sites and cells (Figure 4 and Table 5). Both capillary fraction and local endothelial cell density vary by a single order of magnitude across sites in the mouse brain, whether measured in 2D or 3D (Figure 4A). The two capillary density-related variables are correlated within each structure individually (Table 6), and there is good overlap in data points across structures and animals, especially in the larger 2D dataset (Figure 4A, right), where the overlapping power relationships (Table 6) indicate that the relationship between capillary fraction and cellular composition of capillaries is shared throughout the brain. This agreement suggests that endothelial cell density is a good proxy for the resting rates of blood flow and glucose use (Klein et al., 1986; Noda et al., 2002; Ventura-Antunes et al., 2022).

**TABLE 5:** Spearman correlation coefficients and *p*-values for correlations across cerebral cortical sites.

	D_E_	D_N_	D_*G*_	D_S_	E/N	E/G	E/S	G/N	S/N	S/G
D_E_		–0.084, 0.7047	0.412, 0.0509	–0.194, 0.4133	**0.753,<0.0001**	**0.741,<0.0001**	**0.906,<0.0001**	0.123, 0.5758	–0.166, 0.4837	–0.393, 0.0862
D_N_	–0.084, 0.7047		0.307, 0.1542	*0.460, 0.0414*	**–0.652, 0.0007**	–0.121, 0.5820	–0.235, 0.3177	**–0.624, 0.0015**	**–0.545, 0.0129**	0.056, 0.8113
D_*G*_	0.412, 0.0509	0.307, 0.1542		0.108, 0.6488	0.128, 0.5605	–0.203, 0.3525	0.312, 0.1803	*0.441, 0.0350*	–0.291, 0.2131	**–0.831,<0.0001**
D_S_	–0.194, 0.4133	*0.460, 0.0414*	0.108, 0.6488		–0.441, 0.0517	–0.215, 0.3632	**–0.506, 0.0230**	–0.306, 0.1896	0.404, 0.0775	0.363, 0.1156
E/N	**0.753,<0.0001**	**–0.652, 0.0007**	0.128, 0.5605	–0.441, 0.0517		**0.630, 0.0013**	**0.750, 0.0001**	**0.516, 0.0118**	0.171, 0.4717	–0.306, 0.1893
E/G	**0.741,<0.0001**	–0.121, 0.5820	–0.203, 0.3525	–0.215, 0.3632	**0.630, 0.0013**		**0.628, 0.0030**	–0.273, 0.2069	0.435, 0.0555	0.252, 0.2838
E/S	**0.906,<0.0001**	–0.235, 0.3177	0.312, 0.1803	**–0.506, 0.0230**	**0.750, 0.0001**	**0.628, 0.0030**		0.250, 0.2868	–0.364, 0.1163	**–0.526, 0.0171**
G/N	0.123, 0.5758	**–0.624, 0.0015**	*0.441, 0.0350*	–0.306, 0.1896	**0.516, 0.0118**	–0.273, 0.2069	0.250, 0.2868		0.235, 0.3177	**–0.669, 0.0013**
S/N	–0.166, 0.4837	**–0.545, 0.0129**	–0.291, 0.2131	0.404, 0.0775	0.171, 0.4717	0.435, 0.0555	–0.364, 0.1163	0.235, 0.3177		0.435, 0.0555
S/G	–0.393, 0.0862	0.056, 0.8113	**–0.831,<0.0001**	0.363, 0.1156	–0.306, 0.1893	0.252, 0.2838	**–0.526, 0.0171**	**–0.669, 0.0013**	0.435, 0.0555	

D_E_, local density of endothelial cells; D_N_, local neuronal density; D_G_, local density of glial cells; D_S_, local density of synapses; E/N, ratio of endothelial cells per neuron; E/G, ratio of endothelial cells per glial cell; E/S, ratio of endothelial cells per synapse; G/N, ratio of glial cells per neuron; S/N, ratio of synapses per neuron; S/G, ratio of synapses per glial cell. All values pertaining to D_E_, D_N_ and D_G_ were obtained in the present study, from analysis of 2D images acquired in locations matching those described in Zhu et al. (2018); all values pertaining do D_S_ were calculated from that reference. Values in bold denote significant correlations with p ≤ 0.02. Values in italics denote correlations with p-values between 0.02 and 0.05.

**TABLE 6:** Correlation analysis between variables across sites and animals.

Dependent variable	Independent variable	Structure	N sites	Spearman rho	*P-value*	Exponent	*r* ^2^	*P-value*
Figure 4A								
**Endothelial cells/mm^3^**	**Capillary fraction,%**	**All**	**654**	**0.3450**	**<0.0001**	**0.460 ± 0.046**	**0.135**	**<0.0001**
		**Cx GM**	**72**	**0.3151**	**0.0070**	**0.394 ± 0.137**	**0.106**	**0.0052**
		**Cx WM**	**102**	**0.4118**	**<0.0001**	**0.479 ± 0.124**	**0.130**	**0.0002**
		Hp MolL	55	–0.0015	0.9899	–0.009 ± 0.122	0.000	0.9418
		Hp GranL	9	0.1487	0.3473	0.128 ± 0.175	0.072	0.4864
		**Hypothalamus**	**79**	**0.6101**	**<0.0001**	**0.790 ± 0.134**	**0.311**	**<0.0001**
		**Thalamus**	**121**	**0.5205**	**<0.0001**	**0.652 ± 0.097**	**0.276**	**<0.0001**
		**Striatum**	**102**	**0.3561**	**0.0002**	**0.576 ± 0.126**	**0.172**	**< 0.0001**
		Cb MolL	43	–0.0127	0.9205	0.237 ± 0.183	0.039	0.2015
		Cb GranL	46	0.2976	0.0209	0.086 ± 0.233	0.003	0.7133
		Cb WM	25	–0.0100	0.9404	–0.037 ± 0.185	0.002	0.8421
Figure 4B								
**Endothelial cells/mm^2^**	**Capillary fraction,%**	**All**	**727**	**0.5942**	**<0.0001**	**0.676 ± 0.034**	**0.350**	**<0.0001**
		**Cx GM**	**166**	**0.5882**	**<0.0001**	**0.771 ± 0.068**	**0.439**	**<0.0001**
		**Cx WM**	**100**	**0.6407**	**<0.0001**	**0.728 ± 0.104**	**0.333**	**<0.0001**
		**Hp MolL**	**28**	**0.5348**	**0.0034**	**0.340 ± 0.125**	**0.223**	**0.0112**
		**Hp GranL**	**27**	**0.5107**	**0.0028**	**0.564 ± 0.161**	**0.330**	**0.0017**
		**Hypothalamus**	**31**	**0.6072**	**0.0003**	**0.770 ± 0.195**	**0.349**	**0.0005**
		**Thalamus**	**50**	**0.8044**	**<0.0001**	**0.961 ± 0.093**	**0.691**	**<0.0001**
		**Striatum**	**62**	**0.5044**	**<0.0001**	**0.637 ± 0.126**	**0.300**	**<0.0001**
		**Cb MolL**	**97**	**0.5717**	**<0.0001**	**0.624 ± 0.103**	**0.279**	**<0.0001**
		**Cb GranL**	**99**	**0.2863**	**0.0035**	**0.288 ± 0.121**	**0.055**	**0.0197**
		**Cb WM**	**67**	**0.7388**	**<0.0001**	**0.825 ± 0.086**	**0.585**	**<0.0001**
Figure 4C								
**Neurons/mm^3^**	**Capillary fraction,%**	All	607	0.2726	<0.0001	0.155 ± 0.080	0.006	0.0552
		**Cx GM**	**72**	**0.3041**	**0.0094**	**0.292 ± 0.130**	**0.067**	**0.0280**
		Cx WM	47	0.3068	0.0008	0.368 ± 0.309	0.030	0.2406
		Hp MolL	24	–0.0142	0.9035	0.184 ± 0.170	0.050	0.2907
		Hp GranL	42	0.0494	0.6694	0.241 ± 0.172	0.076	0.1734
		**Hypothalamus**	**79**	**0.2989**	**0.0075**	**0.383 ± 0.126**	**0.107**	**0.0032**
		**Thalamus**	**122**	**0.1946**	**0.0318**	**0.351 ± 0.108**	**0.081**	**0.0015**
		**Striatum**	**102**	**0.2350**	**0.0174**	**0.221 ± 0.087**	**0.060**	**0.0127**
		Cb MolL	57	0.0954	0.4535	0.335 ± 0.232	0.036	0.1544
		Cb GranL	60	–0.1562	0.2334	–0.102 ± 0.110	0.015	0.3537
		Cb WM	25	–0.1637	0.2194	n.a.		
Figure 4D								
**Neurons/mm^2^**	**Capillary fraction,%**	**All**	**628**	**0.2579**	**<0.0001**	**0.464 ± 0.115**	**0.026**	**<0.0001**
		**Cx GM**	**166**	**0.3119**	**<0.0001**	**0.337 ± 0.068**	**0.128**	**<0.0001**
		Cx WM	47	–0.0467	0.6408	0.529 ± 0.356	0.047	0.1436
		Hp MolL	21	–0.0883	0.6552	–0.328 ± 0.316	0.053	0.3128
		Hp GranL	31	–0.0761	0.6790	–0.065 ± 0.104	0.013	0.5396
		Hypothalamus	31	–0.0434	0.8169	0.125 ± 0.184	0.016	0.5038
		**Thalamus**	**50**	**0.3228**	**0.0222**	**0.342 ± 0.143**	**0.107**	**0.0206**
		**Striatum**	**62**	**0.3504**	**0.0052**	**0.446 ± 0.143**	**0.139**	**0.0028**
		Cb MolL	97	0.0835	0.4159	0.059 ± 0.119	0.002	0.6223
		**Cb GranL**	**102**	**–0.3804**	**<0.0001**	**–0.242 ± 0.084**	**0.077**	**0.0047**
		Cb WM	97	0.0835	0.4159	0.059 ± 0.119	0.002	0.6223
Figures 3D, 5A								
**Glia/mm^3^**	**Neurons/mm^3^**	**All**	**528**	**–0.3976**	**<0.0001**	**–0.162 ± 0.024**	**0.080**	**<0.0001**
		Cx GM	72	0.2398	0.0425	0.162 ± 0.107	0.031	0.1368
		Cx WM	47	–0.2684	0.0034	–0.154 ± 0.080	0.077	0.0588
		Hp MolL	22	–0.0575	0.6241	0.252 ± 0.291	0.036	0.3953
		Hp GranL	16	–0.3542	0.0214	0.084 ± 0.519	0.002	0.8729
		Hypothalamus	78	0.1997	0.0776	0.259 ± 0.122	0.056	0.0378
		Thalamus	122	0.1891	0.0370	0.143 ± 0.090	0.021	0.1141
		Striatum	102	0.0844	0.3989	0.050 ± 0.107	0.002	0.6376
		Cb MolL	35	–0.4620	0.0001	0.096 ± 0.154	0.096	0.0705
		**Cb GranL**	**32**	**0.3707**	**0.0035**	**0.959 ± 0.377**	**0.178**	**0.0163**
		Cb WM	35	–0.4620	0.0001	–0.287 ± 0.154	0.096	0.0705
Figures 2D, 5A								
**Glia/mm^2^**	**Neurons/mm^2^**	**All**	**619**	**–0.5202**	**<0.0001**	**–0.257 ± 0.020**	**0.212**	**<0.0001**
		**Cx GM**	**166**	**0.6817**	**<0.0001**	**0.888 ± 0.072**	**0.485**	**<0.0001**
		Cx WM	47	0.2505	0.0111	0.029 ± 0.029	0.022	0.3177
		Hp MolL	21	0.0899	0.6491	0.038 ± 0.120	0.005	0.7529
		Hp GranL	24	–0.2723	0.1316	–0.153 ± 0.332	0.010	0.6397
		Hypothalamus	31	–0.0637	0.7335	–0.097 ± 0.186	0.009	0.6044
		Thalamus	50	–0.1676	0.2446	–0.156 ± 0.122	0.033	0.2082
		**Striatum**	**62**	**0.4281**	**0.0005**	**0.447 ± 0.108**	**0.222**	**0.0001**
		Cb MolL	97	–0.1563	0.1263	–0.222 ± 0.162	0.020	0.1720
		**Cb GranL**	**100**	**0.3934**	**<0.0001**	**1.239 ± 0.176**	**0.336**	**<0.0001**
		Cb WM	21	0.0064	0.9591	0.206 ± 0.093	0.204	0.0399
Figures 3D, 5B								
**Endothelial cells/mm^3^**	**Neurons/mm^3^**	**All**	**530**	**0.1499**	**<0.0001**	**0.132 ± 0.024**	**0.053**	**<0.0001**
		Cx GM	72	0.2430	0.0397	0.149 ± 0.127	0.019	0.2442
		Cx WM	44	0.3494	0.0001	0.011 ± 0.086	0.011	0.4984
		Hp MolL	15	–0.0910	0.4374	–0.132 ± 0.244	0.022	0.5969
		Hp GranL	9	–0.1646	0.2975	–0.050 ± 0.330	0.003	0.8849
		Hypothalamus	79	0.2225	0.0487	0.211 ± 0.136	0.030	0.1239
		Thalamus	121	0.1084	0.2346	0.138 ± 0.092	0.019	0.1343
		**Striatum**	**102**	**0.3594**	**0.0002**	**0.606 ± 0.142**	**0.154**	**<0.0001**
		Cb MolL	40	0.1462	0.2489	0.302 ± 0.101	0.190	0.0049
		Cb GranL	46	0.1482	0.2584	0.261 ± 0.283	0.019	0.3616
		Cb WM	2	n.a.		n.a.		
Figures 2D, 5B								
**Endothelial cells/mm^2^**	**Neurons/mm^2^**	**All**	**620**	**0.1058**	**0.0041**	**0.035 ± 0.016**	**0.008**	**0.0301**
		**Cx GM**	**166**	**0.6777**	**<0.0001**	**0.845 ± 0.070**	**0.467**	**<0.0001**
		Cx WM	46	–0.0097	0.9226	0.378 ± 0.061	0.465	<0.0001
		Hp MolL	21	0.0949	0.6311	0.003 ± 0.114	0.000	0.9818
		Hp GranL	27	–0.4297	0.0141	–0.214 ± 0.270	0.024	0.4372
		Hypothalamus	31	0.1024	0.5835	0.162 ± 0.240	0.015	0.5058
		**Thalamus**	**50**	**0.3409**	**0.0154**	**0.492 ± 0.142**	**0.199**	**0.0012**
		**Striatum**	**62**	**0.4886**	**<0.0001**	**0.399 ± 0.115**	**0.168**	**0.0010**
		Cb MolL	97	0.0017	0.9865	–0.009 ± 0.104	0.000	0.9281
		Cb GranL	99	0.1937	0.0511	0.396 ± 0.131	0.085	0.0033
		Cb WM	21	–0.0364	0.7700	0.187 ± 0.111	0.130	0.1076
Figures 3D, 5C								
**Endothelial cells/mm^3^**	**Glia/mm^3^**	**All**	609	0.0733	0.0394	–0.038 ± 0.042	0.001	0.3719
		Cx GM	72	0.1622	0.1733	0.314 ± 0.135	0.071	0.0233
		Cx WM	102	–0.0497	0.5943	–0.168 ± 0.120	0.019	0.1626
		Hp MolL	49	0.1563	0.1804	0.041 ± 0.103	0.003	0.6895
		Hp GranL	4	0.0782	0.6227	0.500 ± 0.500	0.333	0.4226
		**Hypothalamus**	**78**	**0.4838**	**<0.0001**	**0.496 ± 0.113**	**0.203**	**<0.0001**
		**Thalamus**	**121**	**0.2868**	**0.0014**	**0.342 ± 0.087**	**0.114**	**0.0001**
		Striatum	102	0.1675	0.0924	0.232 ± 0.142	0.026	0.1060
		Cb MolL	31	0.2535	0.0435	–0.032 ± 0.128	0.002	0.8017
		Cb GranL	26	0.1674	0.2010	0.166 ± 0.155	0.046	0.2946
		Cb WM	24	–0.0869	0.5165	0.129 ± 0.130	0.043	0.3316
Figures 2D, 5C								
**Endothelial cells/mm^2^**	**Glia/mm^2^**	**All**	**719**	**0.2327**	**<0.0001**	**0.180 ± 0.025**	**0.069**	**<0.0001**
		**Cx GM**	**166**	**0.6092**	**<0.0001**	**0.607 ± 0.059**	**0.393**	**<0.0001**
		**Cx WM**	**100**	**0.2706**	**0.0060**	**0.463 ± 0.183**	**0.061**	**0.0132**
		Hp MolL	28	0.0958	0.6278	0.189 ± 0.189	0.037	0.3284
		Hp GranL	21	0.1771	0.3322	0.320 ± 0.185	0.136	0.0995
		Hypothalamus	31	0.3218	0.0775	0.434 ± 0.226	0.112	0.0651
		Thalamus	50	0.3048	0.0314	0.315 ± 0.179	0.060	0.0858
		**Striatum**	**62**	**0.4523**	**0.0002**	**0.411 ± 0.121**	**0.161**	**0.0012**
		**Cb MolL**	**97**	**0.4590**	**<0.0001**	**0.250 ± 0.060**	**0.152**	**<0.0001**
		**Cb GranL**	**97**	**0.4635**	**<0.0001**	**0.343 ± 0.055**	**0.292**	**<0.0001**
		**Cb WM**	**67**	**0.2578**	**0.0352**	**0.293 ± 0.138**	**0.065**	**0.0375**
Figure 6A								
**Glia/neuron**	**Neurons/mm^3^**	**All**	**528**	–**0.7611**	**<0.0001**	–**1.162 ± 0.024**	**0.816**	**<0.0001**
		**Cx GM**	**72**	**–0.5422**	**<0.0001**	**–0.838 ± 0.107**	**0.465**	**<0.0001**
		**Cx WM**	**47**	**–0.8862**	**<0.0001**	**–1.154 ± 0.080**	**0.823**	**<0.0001**
		**Hp MolL**	**22**	**–0.5863**	**0.0026**	**–0.747 ± 0.291**	**0.248**	**0.0182**
		Hp GranL	16	–0.4273	0.0048	–0.915 ± 0.519	0.182	0.0996
		**Hypothalamus**	**78**	–**0.5376**	**<0.0001**	**–0.741 ± 0.122**	**0.325**	**<0.0001**
		**Thalamus**	**122**	**–0.6216**	**<0.0001**	**–1.857 ± 0.090**	**0.430**	**<0.0001**
		**Striatum**	**102**	**–0.5799**	**<0.0001**	**–0.949 ± 0.107**	**0.440**	**<0.0001**
		**Cb MolL**	**35**	**–0.5250**	**<0.0001**	**–1.287 ± 0.154**	**0.680**	**<0.0001**
		Cb GranL	32	0.2515	0.0525	–0.041 ± 0.377	0.000	0.9146
		Cb WM	2	n.a.		n.a.		
		**GM structures**	**479**	**–0.7038**	**<0.0001**	**–1.087 ± 0.032**	**0.708**	**<0.0001**
Figure 6C								
**Glia/neuron**	**Glia/mm^3^**	**All**	**528**	**0.7820**	**<0.0001**	**1.495 ± 0.073**	**0.444**	**<0.0001**
		**Cx GM**	**72**	**0.6163**	**<0.0001**	**0.806 ± 0.129**	**0.359**	**<0.0001**
		**Cx WM**	**47**	**0.5395**	**<0.0001**	**1.499 ± 0.257**	**0.430**	**<0.0001**
		**Hp MolL**	**22**	**0.8532**	**<0.0001**	**0.856 ± 0.166**	**0.571**	**<0.0001**
		**Hp GranL**	**16**	**0.9882**	**<0.0001**	**0.978 ± 0.137**	**0.783**	**<0.0001**
		**Hypothalamus**	**78**	**0.6404**	**<0.0001**	**0.786 ± 0.101**	**0.441**	**<0.0001**
		**Thalamus**	**122**	**0.5863**	**<0.0001**	**0.856 ± 0.090**	**0.427**	**<0.0001**
		**Striatum**	**102**	**0.7138**	**<0.0001**	**0.956 ± 0.093**	**0.512**	**<0.0001**
		**Cb MolL**	**35**	**0.9471**	**<0.0001**	**1.333 ± 0.178**	**0.629**	**<0.0001**
		**Cb GranL**	**32**	**0.9816**	**<0.0001**	**0.815 ± 0.073**	**0.807**	**<0.0001**
		Cb WM	2	n.a.		n.a.		
		**GM structures**	**479**	**0.7670**	**<0.0001**	**1.177 ± 0.065**	**0.410**	**<0.0001**
Figure 6E								
**Glia/neuron**	**Endothelial cells/mm^3^**	**All**	**491**	**0.1041**	**0.0103**	**–0.247 ± 0.095**	**0.014**	**0.0096**
		Cx GM	72	0.0362	0.7626	0.098 ± 0.136	0.007	0.4757
		Cx WM	44	–0.1710	0.2505	0.075 ± 0.350	0.001	0.8320
		Hp MolL	14	0.1413	0.5103	1.008 ± 0.412	0.333	0.0307
		Hp GranL	4	0.1012	0.5238	0.938 ± 0.479	0.658	0.1890
		Hypothalamus	78	0.1961	0.0832	0.265 ± 0.119	0.061	0.0293
		Thalamus	121	0.1350	0.1381	0.199 ± 0.117	0.024	0.0928
		Striatum	102	–0.1644	0.0987	–0.143 ± 0.092	0.024	0.1225
		Cb MolL	28	0.2339	0.0799	–0.712 ± 0.460	0.084	0.1339
		Cb GranL	26	0.1136	0.3873	0.44 ± 0.246	0.001	0.8591
		Cb WM	2	n.a.		n.a.		
		GM structures	445	0.1706	<0.0001	–0.012 ± 0.079	0.000	0.8834
Figure 6B								
**Glia/neuron**	**Neurons/mm^2^**	**All**	**619**	**–0.8450**	**<0.0001**	**–1.257 ± 0.020**	**0.865**	**<0.0001**
		Cx GM	166	–0.1281	0.1001	–0.111 ± 0.072	0.014	0.1211
		**Cx WM**	**47**	**–0.9650**	**<0.0001**	**–0.971 ± 0.029**	**0.962**	**<0.0001**
		**Hp MolL**	**21**	**–0.8285**	**<0.0001**	**–0.962 ± 0.120**	**0.771**	**<0.0001**
		**Hp GranL**	**24**	**–0.5765**	**0.0006**	**–1.153 ± 0.322**	**0.368**	**0.0017**
		**Hypothalamus**	**31**	**–0.6972**	**<0.0001**	**–1.097 ± 0.186**	**0.545**	**<0.0001**
		**Thalamus**	**50**	**–0.7806**	**<0.0001**	**–1.156 ± 0.122**	**0.651**	**<0.0001**
		**Striatum**	**62**	**–0.5830**	**<0.0001**	**–0.552 ± 0.108**	**0.303**	**<0.0001**
		**Cb MolL**	**97**	**–0.5924**	**<0.0001**	**–1.222 ± 0.162**	**0.376**	**<0.0001**
		Cb GranL	100	–0.0052	0.9587	0.239 ± 0.176	0.018	0.1769
		**Cb WM**	**21**	–**0.8909**	**<0.0001**	**–0.794 ± 0.093**	**0.792**	**<0.0001**
		**GM structures**	**551**	–**0.7925**	**<0.0001**	**–1.124 ± 0.029**	**0.734**	**<0.0001**
Figure 6D								
**Glia/neuron**	**Glia/mm^2^**	**All**	**619**	**0.7222**	**<0.0001**	**1.824 ± 0.064**	**0.569**	**<0.0001**
		**Cx GM**	**166**	**0.5901**	**<0.0001**	**0.454 ± 0.044**	**0.395**	**<0.0001**
		Cx WM	47	0.1093	0.4646	0.241 ± 0.751	0.002	0.7493
		Hp MolL	21	0.3554	0.1138	0.861 ± 0.436	0.170	0.0630
		**Hp GranL**	**24**	**0.9211**	**<0.0001**	**0.726 ± 0.140**	**0.726**	**<0.0001**
		**Hypothalamus**	**31**	**0.6895**	**<0.0001**	**1.096 ± 0.184**	**0.552**	**<0.0001**
		**Thalamus**	**50**	**0.7051**	**<0.0001**	**1.210 ± 0.165**	**0.528**	**<0.0001**
		**Striatum**	**62**	**0.4399**	**0.0003**	**0.504 ± 0.120**	**0.227**	**<0.0001**
		**Cb MolL**	**97**	**0.8495**	**<0.0001**	**1.088 ± 0.064**	**0.754**	**<0.0001**
		**Cb GranL**	**100**	**0.8797**	**<0.0001**	**0.729 ± 0.038**	**0.785**	**<0.0001**
		Cb WM	21	–0.0494	0.8318	0.010 ± 0.449	0.000	0.9816
		**GM structures**	**551**	**0.6442**	**<0.0001**	**1.263 ± 0.061**	**0.438**	**<0.0001**
Figure 6F								
**Glia/neuron**	**Endothelial cells/mm^2^**	**All**	**612**	**0.1768**	**<0.0001**	0.302 ± 0.134	0.008	0.0247
		Cx GM	166	0.0765	0.3273	0.094 ± 0.058	0.016	0.1051
		**Cx WM**	**46**	**–0.1882**	**0.2052**	**–1.080 ± 0.214**	**0.367**	**<0.0001**
		Hp MolL	21	0.0825	0.7221	0.231 ± 0.503	0.011	0.6517
		Hp GranL	21	0.2869	0.1113	0.571 ± 0.303	0.158	0.0748
		Hypothalamus	31	0.1565	0.4006	0.164 ± 0.210	0.021	0.4405
		Thalamus	50	–0.0167	0.9086	–0.213 ± 0.185	0.027	0.2545
		Striatum	62	–0.0528	0.6838	–0.029 ± 0.133	0.001	0.8258
		**Cb MolL**	**97**	**0.3234**	**0.0012**	**0.620 ± 0.191**	**0.100**	**0.0016**
		**Cb GranL**	**97**	**0.4672**	**<0.0001**	**0.641 ± 0.114**	**0.250**	**<0.0001**
		Cb WM	21	–0.2117	0.3570	–0.426 ± 0.383	0.061	0.2793
		**GM structures**	**545**	**0.3382**	**<0.0001**	**0.998 ± 0.093**	**0.174**	**<0.0001**
Figure 7A								
**Endothelial cells/neuron**	**Neurons/mm^3^**	**All**	**530**	**–0.6724**	**<0.0001**	**–0.868 ± 0.024**	**0.706**	**<0.0001**
		**Cx GM**	**72**	**–0.4863**	**<0.0001**	**–0.851 ± 0.127**	**0.390**	**<0.0001**
		**Cx WM**	**44**	**–0.6024**	**<0.0001**	**–1.058 ± 0.086**	**0.784**	**<0.0001**
		**Hp MolL**	15	–0.0667	0.7567	**–1.132 ± 0.244**	**0.624**	**0.0005**
		Hp GranL	9	–0.2148	0.1719	–1.050 ± 0.330	0.591	0.0155
		**Hypothalamus**	**79**	**–0.5261**	**<0.0001**	**–0.788 ± 0.136**	**0.304**	**<0.0001**
		**Thalamus**	**121**	**–0.6059**	**<0.0001**	**–0.862 ± 0.092**	**0.426**	**<0.0001**
		**Striatum**	**102**	**–0.3124**	**0.0014**	**–0.394 ± 0.142**	**0.072**	**0.0065**
		**Cb MolL**	**40**	**–0.4594**	**0.0003**	**–0.698 ± 0.101**	**0.556**	**<0.0001**
		Cb GranL	46	–0.1465	0.2642	–0.739 ± 0.283	0.134	0.0122
		Cb WM	2	n.a.		n.a.		
		**GM structures**	**484**	–**0.6379**	**<0.0001**	–**0.879 ± 0.032**	**0.612**	**<0.0001**
Figure 7C								
**Endothelial cells/neuron**	**Glia/mm^3^**	**All**	**491**	**0.4007**	**<0.0001**	**0.521 ± 0.078**	**0.083**	**<0.0001**
		Cx GM	72	0.0621	0.6045	0.120 ± 0.178	0.006	0.5007
		Cx WM	44	–0.0228	0.8794	0.371 ± 0.327	0.030	0.2627
		Hp MolL	14	0.2353	0.2683	0.119 ± 0.382	0.008	0.7601
		Hp GranL	4	0.0957	0.5464	0.244 ± 0.898	0.036	0.8109
		Hypothalamus	78	0.2155	0.0565	0.282 ± 0.146	0.047	0.0568
		Thalamus	121	0.0983	0.2815	0.197 ± 0.120	0.022	0.1024
		Striatum	102	0.1512	0.1293	0.188 ± 0.136	0.019	0.1710
		Cb MolL	28	0.4360	0.0007	0.354 ± 0.194	0.114	0.0785
		Cb GranL	26	0.0595	0.6515	0.028 ± 0.141	0.002	0.8419
		Cb WM	2	n.a.		n.a.		
		**GM structures**	**445**	**0.3614**	**<0.0001**	**0.304 ± 0.075**	**0.036**	**<0.0001**
Figure 7E								
**Endothelial cells/neuron**	**Endothelial cells/mm^3^**	**All**	**530**	**0.5915**	**<0.0001**	**0.600 ± 0.074**	**0.112**	**<0.0001**
		**Cx GM**	**72**	**0.6810**	**<0.0001**	**0.870 ± 0.110**	**0.471**	**<0.0001**
		**Cx WM**	**44**	**0.6229**	**<0.0001**	**1.188 ± 0.274**	**0.308**	**<0.0001**
		**Hp MolL**	**15**	**0.9729**	**<0.0001**	**1.167 ± 0.309**	**0.524**	**0.0023**
		**Hp GranL**	**9**	**0.9942**	**<0.0001**	**1.065 ± 0.431**	**0.466**	**0.0429**
		**Hypothalamus**	**79**	**0.6696**	**<0.0001**	**0.856 ± 0.093**	**0.526**	**<0.0001**
		**Thalamus**	**121**	**0.6790**	**<0.0001**	**0.864 ± 0.090**	**0.437**	**<0.0001**
		**Striatum**	**102**	**0.7182**	**<0.0001**	**0.745 ± 0.060**	**0.610**	**<0.0001**
		Cb MolL	40	0.7711	<0.0001	0.371 ± 0.211	0.076	0.0860
		**Cb GranL**	**46**	**0.9230**	**<0.0001**	**0.927 ± 0.079**	**0.759**	**<0.0001**
		Cb WM	2	n.a.		n.a.		
		**GM structures**	**484**	**0.6704**	**<0.0001**	**0.760 ± 0.063**	**0.231**	**<0.0001**
Figure 7B								
**Endothelial cells/neuron**	**Neurons/mm^2^**	**All**	**620**	**–0.8553**	**<0.0001**	**–0.965 ± 0.016**	**0.853**	**<0.0002**
		**Cx GM**	**166**	**–0.1877**	**0.0155**	**–0.155 ± 0.070**	**0.029**	**0.0290**
		**Cx WM**	**46**	**–0.8327**	**<0.0002**	**–0.621 ± 0.061**	**0.701**	**<0.0001**
		**Hp MolL**	**21**	**–0.8893**	**<0.0001**	**–0.997 ± 0.114**	**0.802**	**<0.0001**
		**Hp GranL**	**27**	**–0.7353**	**<0.0001**	**–1.214 ± 0.270**	**0.446**	**0.0001**
		**Hypothalamus**	**31**	**–0.6492**	**<0.0001**	**–0.838 ± 0.240**	**0.296**	**0.0016**
		**Thalamus**	**50**	**–0.5413**	**<0.0001**	**–0.508 ± 0.142**	**0.209**	**0.0008**
		**Striatum**	**62**	**–0.5308**	**<0.0001**	**–0.601 ± 0.115**	**0.314**	**<0.0001**
		**Cb MolL**	**97**	**–0.6847**	**<0.0001**	**–1.009 ± 0.104**	**0.496**	**<0.0001**
		**Cb GranL**	**99**	**–0.4014**	**<0.0001**	**–0.604 ± 0.131**	**0.179**	**<0.0001**
		**Cb WM**	**21**	**–0.8388**	**<0.0001**	**–0.813 ± 0.111**	**0.739**	**<0.0001**
		**GM structures**	**553**	**–0.8243**	**<0.0001**	**–1.082 ± 0.022**	**0.805**	**<0.0001**
Figure 7D								
**Endothelial cells/neuron**	**Glia/mm^2^**	**All**	**612**	**0.5240**	**<0.0001**	**1.072 ± 0.062**	**0.332**	**<0.0001**
		Cx GM	166	0.0492	0.5291	0.062 ± 0.056	0.007	0.2722
		Cx WM	46	0.1352	0.3647	0.420 ± 0.572	0.012	0.4666
		Hp MolL	21	–0.0007	0.9978	0.077 ± 0.486	0.001	0.8765
		Hp GranL	21	0.2199	0.2266	0.423 ± 0.245	0.136	0.1000
		Hypothalamus	31	0.3673	0.0421	0.530 ± 0.266	0.120	0.0562
		**Thalamus**	**50**	**0.3911**	**0.0050**	**0.525 ± 0.170**	**0.166**	**0.0034**
		Striatum	62	–0.0672	0.6037	–0.085 ± 0.145	0.006	0.5618
		**Cb MolL**	**97**	**0.4094**	**<0.0001**	**0.337 ± 0.086**	**0.140**	**0.0002**
		Cb GranL	97	0.1279	0.2001	0.066 ± 0.069	0.010	0.3385
		Cb WM	21	–0.3355	0.1371	–0.640 ± 0.452	0.095	0.1730
		**GM structures**	**545**	**0.3867**	**<0.0001**	**0.683 ± 0.069**	**0.153**	**<0.0001**
Figure 7F								
**Endothelial cells/neuron**	**Endothelial cells/mm^2^**	**All**	**620**	**0.3497**	**<0.0001**	**0.783 ± 0.100**	**0.091**	**<0.0001**
		**Cx GM**	**166**	**0.5384**	**<0.0001**	**0.447 ± 0.046**	**0.364**	**<0.0001**
		Cx WM	46	0.1956	0.1875	–0.230 ± 0.199	0.029	0.2541
		Hp MolL	21	0.3531	0.1164	0.989 ± 0.462	0.194	0.0455
		**Hp GranL**	**27**	**0.9011**	**<0.0001**	**1.114 ± 0.144**	**0.704**	**<0.0001**
		**Hypothalamus**	**31**	**0.6278**	**0.0002**	**0.905 ± 0.141**	**0.585**	**<0.0001**
		**Thalamus**	**50**	**0.5568**	**<0.0001**	**0.595 ± 0.117**	**0.350**	**<0.0001**
		**Striatum**	**62**	**0.4069**	**0.0010**	**0.580 ± 0.121**	**0.277**	**<0.0001**
		**Cb MolL**	**97**	**0.6868**	**<0.0001**	**1.009 ± 0.101**	**0.513**	**<0.0001**
		**Cb GranL**	**99**	**0.7647**	**<0.0001**	**0.784 ± 0.072**	**0.552**	**<0.0001**
		Cb WM	21	0.1443	0.5325	0.303 ± 0.413	0.027	0.4726
		**GM structures**	**553**	**0.5258**	**<0.0001**	**1.283 ± 0.078**	**0.329**	**<0.0001**
Figure 8								
**Endothelial cells/neuron**	**Glia/neuron (3D)**	**All**	**491**	**0.6371**	**<0.0001**	**0.645 ± 0.023**	**0.609**	**<0.0001**
		**Cx GM**	**72**	**0.4862**	**<0.0001**	**0.629 ± 0.109**	**0.323**	**<0.0001**
		**Cx WM**	**44**	**0.4673**	**0.0009**	**0.729 ± 0.092**	**0.599**	**<0.0001**
		Hp MolL	14	0.2512	0.2363	0.785 ± 0.215	0.526	0.0033
		Hp GranL	4	0.1230	0.4379	0.959 ± 0.610	0.552	0.2567
		**Hypothalamus**	**78**	**0.5360**	**<0.0001**	**0.668 ± 0.100**	**0.369**	**<0.0001**
		**Thalamus**	**121**	**0.5480**	**<0.0001**	**0.620 ± 0.073**	**0.376**	**<0.0001**
		**Striatum**	**102**	**0.2816**	**0.0041**	**0.298 ± 0.098**	**0.084**	**0.0032**
		**Cb MolL**	**28**	**0.4601**	**0.0003**	**0.424 ± 0.084**	**0.493**	**<0.0001**
		Cb GranL	26	0.0474	0.7190	0.050 ± 0.150	0.004	0.7425
		Cb WM	2	n.a.		n.a.		
		**GM structures**	**445**	**0.5986**	**<0.0001**	**0.635 ± 0.031**	**0.482**	**<0.0001**
**Endothelial cells/neuron**	**Glia/neuron (2D)**	**All**	**612**	**0.8538**	**<0.0001**	**0.714 ± 0.012**	**0.849**	**<0.0001**
		**Cx GM**	**166**	**0.2679**	**0.0005**	**0.300 ± 0.074**	**0.091**	**<0.0001**
		**Cx WM**	**46**	**0.8774**	**<0.0001**	**0.652 ± 0.056**	**0.755**	**<0.0001**
		**Hp MolL**	**21**	**0.8419**	**<0.0001**	**0.850 ± 0.128**	**0.697**	**<0.0001**
		**Hp GranL**	**21**	**0.4367**	**0.0124**	**0.566 ± 0.166**	**0.378**	**0.0030**
		**Hypothalamus**	**31**	**0.7157**	**<0.0001**	**0.623 ± 0.154**	**0.361**	**0.0004**
		**Thalamus**	**50**	**0.6504**	**<0.0001**	**0.437 ± 0.092**	**0.318**	**<0.0001**
		**Striatum**	**62**	**0.4926**	**<0.0001**	**0.521 ± 0.120**	**0.237**	**<0.0001**
		**Cb MolL**	**97**	**0.6260**	**<0.0001**	**0.469 ± 0.056**	**0.425**	**<0.0001**
		**Cb GranL**	**97**	**0.3471**	**0.0004**	**0.303 ± 0.079**	**0.134**	**0.0002**
		**Cb WM**	**21**	**0.8004**	**<0.0001**	**0.854 ± 0.144**	**0.649**	**<0.0001**
		**GM structures**	**545**	**0.6115**	**<0.0001**	**0.823 ± 0.018**	**0.789**	**<0.0001**
Figure 9								
**Endothelial cells/glia**	**Endothelial cells/mm^2^**	**Cx GM**	**23**	**0.741**	**<0.0001**	**0.801 ± 0.158**	**0.551**	**<0.0001**
**Endothelial cells/glia**	**Endothelial cells/neuron**	**Cx GM**	**23**	**0.630**	**0.0013**	**0.707 ± 0.146**	**0.528**	**<0.0001**
**Endothelial cells/glia**	**Endothelial cells/synapse**	**Cx GM**	**23**	**0.628**	**0.0030**	**0.622 ± 0.167**	**0.435**	**0.0016**
**Endothelial cells/neuron**	**Endothelial cells/mm^2^**	**Cx GM**	**23**	**0.753**	**<0.0001**	**0.862 ± 0.152**	**0.604**	**<0.0001**
**Endothelial cells/neuron**	**Endothelial cells/synapse**	**Cx GM**	**23**	**0.750**	**0.0001**	**0.807 ± 0.121**	**0.712**	**<0.0001**
**Endothelial cells/neuron**	**Glia/neuron**	**Cx GM**	**23**	**0.5156**	**0.0118**	0.550 ± 0.274	0.161	0.0577
**Endothelial cells/synapse**	**Endothelial cells/mm^2^**	**Cx GM**	**23**	**0.906**	**<0.0001**	**1.117 ± 0.109**	**0.854**	**<0.0001**
**Endothelial cells/synapse**	**Endothelial cells/neuron**	**Cx GM**	**23**	**0.750**	**<0.0001**	**0.882 ± 0.132**	**0.712**	**<0.0001**
**Endothelial cells/synapse**	**Synapses/mm^2^**	**Cx GM**	**23**	**–0.506**	**0.0230**	**–1.515 ± 0.478**	**0.358**	**0.0053**
**Glia/mm^2^**	**Synapses/glia**	**Cx GM**	**23**	**–0.831**	**<0.0001**	**–0.784 ± 0.104**	**0.759**	**<0.0001**
**Glia/neuron**	**Endothelial cells/neuron**	**Cx GM**	**23**	**0.516**	**0.0118**	0.293 ± 0.146	0.161	0.0577
**Glia/neuron**	**Glia/mm^2^**	**Cx GM**	**23**	**0.441**	**0.0350**	**0.634 ± 0.191**	**0.344**	**0.0032**
**Glia/neuron**	**Neurons/mm^2^**	**Cx GM**	**23**	**–0.624**	**0.0015**	**–0.592 ± 0.212**	**0.270**	**0.0110**
**Glia/neuron**	**Synapses/glia**	**Cx GM**	**23**	**–0.669**	**0.0013**	**–0.688 ± 0.143**	**0.562**	**0.0001**
**Neurons/mm^2^**	**Synapses/mm^2^**	**Cx GM**	**23**	**0.460**	**0.0414**	0.654 ± 0.317	0.191	0.0541
**Neurons/mm^2^**	**Synapses/neuron**	**Cx GM**	**23**	**–0.545**	**0.0129**	**–0.820 ± 0.164**	**0.581**	**<0.0001**
**Synapses/glia**	**Endothelial cells/synapse**	**Cx GM**	**23**	**–0.526**	**0.0171**	**–0.378 ± 0.167**	**0.222**	**0.0361**
**Synapses/glia**	**Glia/mm^2^**	**Cx GM**	**23**	**–0.831**	**<0.0001**	**–0.969 ± 0.129**	**0.759**	**<0.0001**
**Synapses/glia**	**Glia/neuron**	**Cx GM**	**23**	**–0.669**	**0.0013**	**–0.816 ± 0.170**	**0.562**	**0.0001**
**Synapses/neuron**	**Neurons/mm^2^**	**Cx GM**	**23**	**–0.545**	**0.0129**	**–0.708 ± 0.142**	**0.581**	**<0.0001**
**Synapses/neuron**	**Synapses/mm^2^**	**Cx GM**	**23**	**0.750**	**0.0001**	0.346 ± 0.317	0.062	0.2890

Lines in bold denote structures with both a significant correlation and a significant power scaling between the variables at the level of p < 0.05, except for correlations in Figure 9, where all relationships with p < 0.05 are indicated in bold.

Neuronal densities, in contrast, vary over two orders of magnitude across sites in GM structures, from 31,250 to 3,843,750/mm^3^ (Figure 4B), and are not significantly correlated with local capillary fraction (Figure 4B) across brain sites and structures (Table 6). In fact, brain structures with enormously different neuronal densities have capillary fractions in the same restricted range (Figure 4B), and correlations, where significant, can be positive (cortical GM) or negative (cerebellar granular layer; Table 6).

**FIGURE 5:**
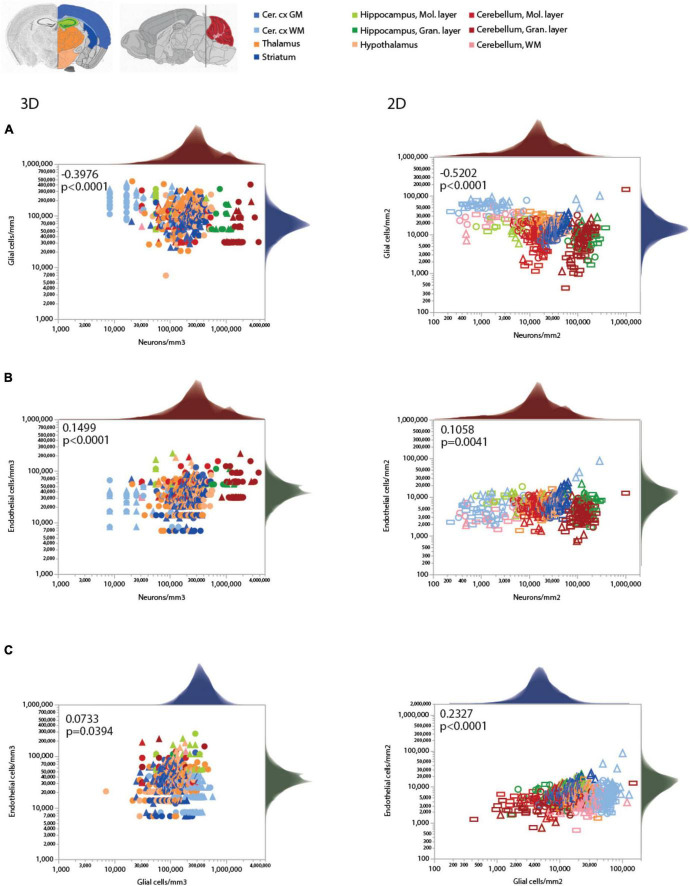
Local densities of different cell types vary concertedly only within some brain structures. Each plot is accompanied by the respective distribution histograms aligned with the X and Y axes. Spearman correlation coefficients and *p*-values across all structures and sites are indicated in each graph. **(A)** Glial cell densities span just over one order of magnitude across structures, while neuronal cell densities span two orders of magnitude. While the correlation across all data points is significant and negative in both 3D and 2D datasets, analysis at that level ignores obvious differences across structures (see Table 6). **(B)** Similarly, endothelial cell densities are concentrated within one order of magnitude across structures and sites, and are similar across structures with very different neuronal densities. Still, local endothelial and neuronal cell densities are significantly and positively correlated within the cortical gray matter, the thalamus, and the striatum (see Table 6). **(C)** Glial and endothelial cell densities, which vary little across sites and structures in comparison to neuronal densities, are not strongly correlated across brain structures, and weakly correlated within some (see Table 6). All graphs are shown with similar X and Y scales for comparison.

Like endothelial cell densities, variation in glial cell densities is restricted to within one order of magnitude across sites in the mouse brain (Figure 5A). Within the cortical GM, local glial cell densities vary significantly in positive correlation with local neuronal density, as previously observed across sites within both human (Lauwers et al., 2008) and mouse cortices (Herculano-Houzel et al., 2013; Figure 5A and Table 6). In contrast, there is no universal correlation between local neuronal and glial cell densities across brain structures; if anything, there is a modest negative correlation across all sites (Figure 5A), which is due to the slightly more elevated glial cell densities in WM sites.

Importantly, a large range of neuronal densities occurs with similar endothelial cell densities across structures in the mouse brain, with only a weak overall trend toward higher endothelial cell densities where neuronal densities are higher (Figure 5B). A significant increase in local endothelial cell density in sites with increasing local neuronal density is found only in the cortical GM, thalamus and striatum (Figure 5B and Table 6). Similarly, we find significant correlations between local densities of endothelial cells and glial cells within some structures, but no strong systematic correlation across locations (Figure 5C).

**FIGURE 6:**
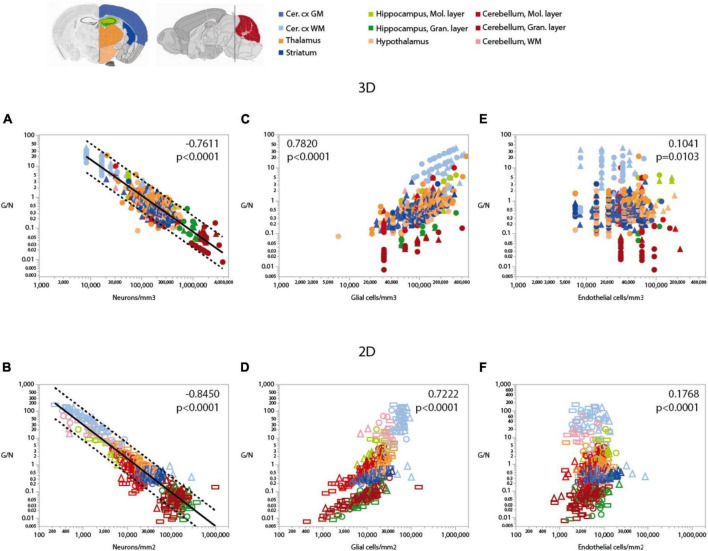
Ratio of glial cells per neuron (G/N) increases across brain structures and sites with decreasing neuronal densities. Spearman correlation coefficients and *p*-values across all structures and sites are indicated in each graph; relationships for each structure are given in Table 6. G/N ratios span over three orders of magnitude and vary as a single power function of neuronal density across all brain structures and sites whether measured in 3D stacks **(A)** or in 2D composite images **(B)**. Power functions are plotted with 95% confidence interval (dashed lines), and have exponents –1.162 ± 0.024, *r*^2^ = 0.816, *p* < 0.0001 (**A**, 3D) or –1.257 ± 0.020, *r*^2^ = 0.865, *p* < 0.0001 (**B**, 2D). Notice that most data points in all structures are contained within the 95% CI of the function that applies across structures, indicating that the relationship is brain-wide. **(C,D)** G/N ratio also correlates with glial cell densities across all brain structures and sites, but the relationships are clearly distinct across sites (see exponents in Table 5). **(E,F)** Although a weak correlation is detected between G/N ratios and endothelial cell densities across brain structures and sites, most local correlations are non-significant or weak, with a wide range of non-overlapping values of G/N for similar endothelial cell densities across brain structures. All graphs are shown with similar X and Y scales for comparison.

Consistently with the larger variation of neuronal densities than glial cell densities across locations, the glia/neuron (G/N) ratio is highly variable within the mouse brain, and is universally and inversely related to local neuronal densities across all sites and structures, as we described previously (Mota and Herculano-Houzel, 2014; Figures 6A,B). That is, there are more glial cells per neuron in those sites with lower neuronal densities, in a relationship that applies both across and within structures, regardless of their identity (Table 6). In contrast, the relationship between local glia/neuron ratio and glial cell density is specific to each structure, with different ranges of glia/neuron ratios sharing similar glial cell densities across structures (Figures 6C,D). Importantly, although higher G/N ratios where neuronal densities are smaller (and therefore neurons are larger) have been proposed to accompany the presumably higher energetic demands of larger neurons (Attwell and Laughlin, 2001), the entire 10,000-fold range of G/N ratios occurs within the same restricted range of endothelial cell densities, with no consistent correlation across brain structures and sites (Figures 6E,F). That is, endothelial cell densities are not significantly higher in those locations where each neuron is accompanied by a larger number of glial cells.

**FIGURE 7:**
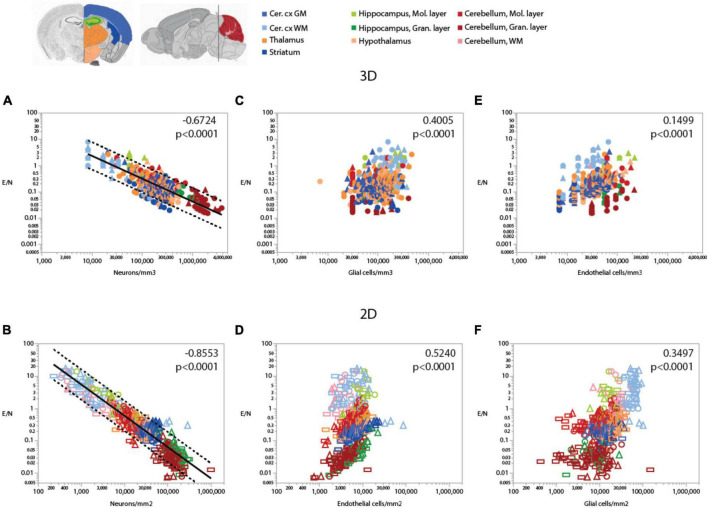
Ratio of endothelial cells per neuron (E/N) varies widely and uniformly across brain structures and sites depending on local neuronal density, but correlations with glial and endothelial cell densities only apply locally. Spearman correlation coefficients and *p*-values across all structures and sites are indicated in each graph; correlations and power function exponents for each structure are listed in Table 4. **(A,B)** E/N ratios span three orders of magnitude across brain sites and structures and vary as a single power function of local neuronal density whether measured in 3D stacks **(A**) or in 2D composite images **(B)**. **(C,D)** E/N ratio appears to correlate with local glial cell density across brain structures in both datasets, but closer inspection reveals that the overall correlation results from the combination of locations and does not hold within most structures (see Table 3). **(E,F)** In contrast, E/N ratios are strongly correlated with endothelial cell densities locally within each structure, but different brain structures have non-overlapping E/N ratios with similar endothelial cell densities, indicating that the apparent overall correlation does not result from a universal correlation, unlike that seen for the strong and universal correlation between E/N and neuronal density **(A,B)**. All graphs are shown with similar X and Y scales for comparison.

**FIGURE 8:**
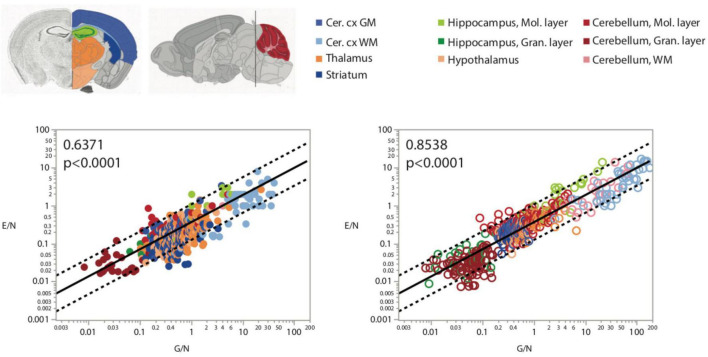
More endothelial cells per neuron (E/N) are uniformly found in brain structures and sites with more glial cells per neuron, as expected given that both these variables are strongly and inversely correlated with local neuronal density (Figures 2, 3). Spearman correlation coefficients are indicated in the graphs.

Because of the much larger variation in neuronal densities than in endothelial cell densities across sites, the local ratio of endothelial cells per neuron is inversely proportional to local neuronal density across all sites and structures examined (Figure 7). Importantly, this relationship is universal across brain locations, as the power function that applies across structures contains all structures and almost all data points within its 95% CI (Figures 7A,B). That is, each neuron is accompanied by more endothelial cells at sites with lower neuronal densities than at sites with higher neuronal densities. In contrast, the ratio of endothelial cells per neuron does not vary consistently with local glial cell density neither across nor within brain structures (Figures 7C,D and Table 6). As expected for the quantity in the numerator of the ratio, local endothelial cell density is very strongly correlated with the E/N ratio in each brain structure in the mouse brain, but there is hardly any overlap across structures: because of the widely different neuronal densities across structures, brain sites with similar densities of endothelial cells have very different E/N ratios across structures (Figures 7E,F). As could be expected of two quantities that are heavily dependent on variation in neuronal density, the E/N ratio is strongly and linearly correlated with the G/N ratio across all sites and structures (Figure 8). That is, where more endothelial cells supply energy per neuron (i.e., in structures and sites with low neuron density), there are also more glial cells per neuron. Importantly, the relationship that applies across structures includes most data points for every structure, indicating that this relationship is universal.

**FIGURE 9:**
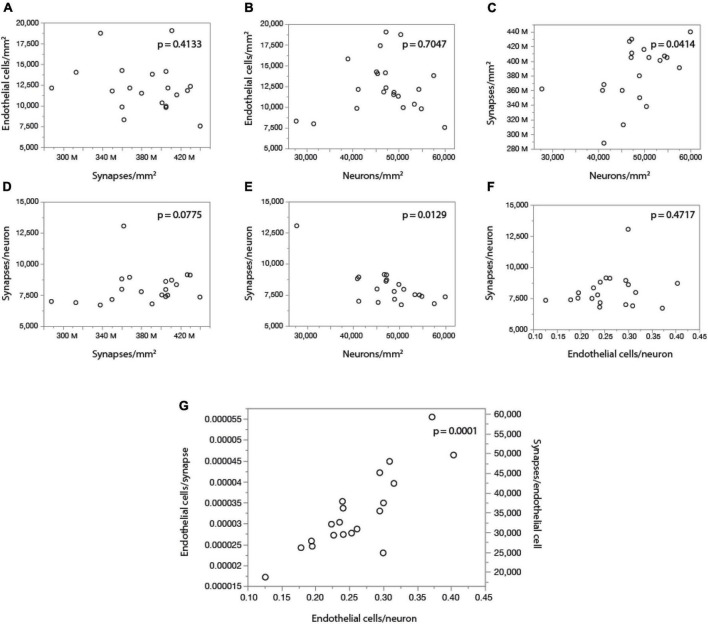
With fairly constant numbers of synapses per neuron, the number of synapses served per endothelial cell increases together with the ratio of endothelial cells per neuron across sites in the mouse cerebral cortex. Data on local synaptic densities from Zhu et al. (2018) were combined to estimates of local densities of endothelial cells, neurons and glial cells in 2D images acquired in matching locations of the cerebral cortex as described in Zhu et al. (2018). Local densities of endothelial cells are not significantly correlated with local synaptic densities **(A)** or neuronal densities **(B)**. Sites with more neurons have marginally, but significantly, more synapses **(C)**, which is consistent with a lack of correlation between local synaptic density and the number of synapses per neuron **(D)**, although a correlation between average number of synapses per neuron and local neuronal density, due to one outlier, cannot be discarded **(E)**. Still, there is very clearly no correlation between local numbers of synapses per neuron and the ratio of endothelial cells per neuron **(F)**; rather, the latter is directly, and strongly (*r*^2^ = 0.750), correlated with the ratio of endothelial cells per synapse (or its inverse, the number of synapses per endothelial cell; **G)**. All correlation coefficients are available in Table 6.

Finally, by comparing our measurements of local cell densities and capillary fraction in cortical gray matter sites matching those with recently published data on local synaptic densities in the mouse cerebral cortex (Zhu et al., 2018), we could examine whether there is evidence of higher energy availability in sites of higher synaptic densities, even at rest, to support the expectation that energy supply responds to local demands due to synaptic activity by self-reorganization of capillary densities (Attwell and Laughlin, 2001; Harris et al., 2012). Densities of synapses (measured as synapses that express PSD and/or SAP according to data in Zhu et al., 2018) vary 1.5-fold across cortical sites, while densities of endothelial cells vary 2.5-fold and neuronal densities vary a similar 2.2-fold. However, we find no significant correlation between local endothelial cell density and synaptic density (Figure 9A) or neuronal density (Figure 9B) across areas within the mouse cerebral cortex (Spearman, *p* = 0.4133 and 0.7047), indicating that cortical areas with more synapses or more neurons receive as much capillary supply as sites with fewer synapses or neurons. Higher local synaptic densities are only modestly associated with higher local neuronal densities (Figure 9C; Spearman, *p* = 0.0414), such that the ratio of synapses per neuron is concentrated in the 6,500–9,500 range (Figure 9E; Spearman, *p* = 0.0129 due to a single data point above 12,500), compatible with previous estimates of ca. 8,000 synapses per cortical neuron in the mouse (Schüz and Palm, 1989; Braitenberg and Schüz, 1998). We find that the average number of synapses per neuron is not higher where there are more synapses (Figure 9D; Spearman, *p* = 0.0775), and is also not significantly correlated with the local number of glial cells per neuron (Spearman, *p* = 0.3177; Table 6). Indeed, local densities of synapses are not correlated with local glial cell densities (Spearman, *p* = 0.6488), and the higher the local glial cell density, the fewer the synapses per glial cell (Spearman rho = -0.831, *p* < 0.0001; Table 6). Crucially, the average number of synapses per neuron is not significantly correlated with the local ratio of endothelial cells per neuron (Figure 9F; Spearman, *p* = 0.4717): the E/N ratio varies about four-fold across cortical sites with similar average numbers of synapses per neuron, and at a similar capillary supply per neuron, local neurons may have variable average numbers of synapses. Instead, the most striking pattern is, again, that at cortical sites where each neuron has more capillary cells available to it, there are also more capillary cells available per synapse (E/S; Figure 9G; Spearman, *p* = 0.0001).

These data thus indicate that higher E/S is associated with higher E/N, even though there are not more endothelial cells where there are more synapses (or more neurons; Figures 9A,B), and there are also not more E/N where there are more S/N (Figure 9F). Rather, higher E/S occurs with higher E/N (Figure 9G) simply due to small variations in endothelial cell densities that are not correlated with variations in densities of neurons or synapses (Figures 9A,B) in the presence of significant local variations in neuronal densities that are only somewhat correlated with smaller variations in densities of synapses (Figure 9C). A principal component analysis supports this conclusion. We find that three factors are required to account for the variance in all six variables, with capillary density loading only in the first factor [explaining 42.9% of the variance; factor loading, 0.996, associated with E/N (0.816) and E/S (0.926)], neuronal density loading only in the second factor [total variance explained, 78.8%; loading, –0.943, associated with E/N (0.557) and S/N (0.976)], and synaptic density as the sole contributor to the third factor (explaining a total of 99.3% of the variance; factor loading, 0.981).

Finally, while the glia/neuron ratio is locally higher in cortical sites where the endothelial cell/neuron ratio is also higher (Spearman, *p* = 0.0118), the number of endothelial cells per synapse is not correlated with the local glia/neuron ratio (*p* = 0.2868; Table 6). Thus, a higher proportion of glial cells per neuron is not associated with a larger capillary supply per synapse.

## Discussion

The brain is an expensive organ and, at rest, already uses nearly as much energy as during sensory activation (Leithner and Royl, 2014). The high energy cost of the brain has traditionally been regarded as driven by an unusually high energy demand by neurons, leading to the expectation that sites with more neurons, or larger neurons, or more synapses use more energy and thus have more capillary supply. Instead, here we find that capillary density is relatively homogeneous across brain structures despite a 100-fold variation in local neuronal densities. We propose that in the adult steady-state measured in a fixed brain, neurons are constrained to using what energy is made available by the relatively homogeneous capillary supply, with little evidence for plastic, self-organizing adjustments according to local demand (see Herculano-Houzel and Rothman, 2022). Such a supply-limited view of brain metabolism explains its high risk of ischemia and vulnerability to states of compromised metabolism, including normal aging.

While neuroscientists often frame the energy *cost* of the brain in terms of high neuronal *demand* for energy, we prefer to reframe metabolism simply as energy *use*, a term that implies nothing about what defines it, whether supply or demand, and instead leaves these open to investigation (Herculano-Houzel and Rothman, 2022). The issue of energetic *use* by neurons can then be separated into the more tractable questions of (a) whether there is evidence that larger neurons *demand* and thus are provided with more energy, depending on their intrinsic biophysical properties (Lin et al., 2010), and (b) whether there is evidence that larger neurons are *supplied* with more energy than smaller neurons, regardless of (a). Our finding that local capillary area fraction or density of capillary cells does not accompany the enormous variation in neuronal densities across sites in the mouse brain nor synaptic densities in the mouse cerebral cortex provides evidence against (a) and in support of (b), against several intuitive, but so far untested, central tenets of neurophysiology and functional brain imaging. These untested tenets are, namely: that neurons *demand* energy, and larger neurons *demand* (and receive) more energy than smaller neurons; that sites with more synapses use more energy; that because larger neurons have more synapses, they demand (and receive) more energy; and that the steady-state density of the adult capillary bed reflects self-organized adjustments across brain sites according to variations in local energy demand by neurons and synapses.

Instead, given our main finding of a comparatively constant capillary density, we interpret our data to indicate that, with the 100× larger variation in neuronal densities throughout the mouse brain, larger neurons *get access* to more energy simply because where neurons are larger, as indicated by lower neuronal densities, fewer neurons compete for the capillary density-restricted blood supply. Although neuronal networks are by definition distributed, thanks to the far-reaching dendrites and axons that define neurons, the neuronal cell body is the site of massive macromolecule synthesis that constitutes a major destination of resources; thus, the E/N ratio, defined here as the number of capillary cells supplying an individual neuronal cell body, is a physiologically relevant variable, although individual neurons will undoubtedly receive partial energetic support from other capillary cells that supply territories where their neurites extend.

Similarly to larger neurons having less competition for blood supply that is limited by capillary density, and given the small but significant variation in densities of synapses with densities of neurons within the cerebral cortex, we propose that there is a restricted range of numbers of synapses per neuron, and, where neurons are larger, more endothelial cells provide energy both per neuron and per synapse (highlighted middle panel in Figure 3). These findings suggest the intriguing possibility that, whereas cell sizes in mammalian bodies are constant across species for all other cell types, increasing neuronal sizes in evolution may have brought neurons the advantage of *not* decreasing energy availability per neuronal unit (with its surrounding glial cells), and possibly holding that constant, as body size increases and total metabolic rate scales more slowly than body mass (Herculano-Houzel, 2011), a possibility that we are now ready to address by extending the present analysis to other species.

Within the cerebral cortex, in particular, a previous detailed study of variation in fractional volume of the microvasculature across layers found that it varies little, and does not reflect variations in neuronal density across cortical layers (Tsai et al., 2009). When compiling data across layers, that study did find a significant positive correlation between neuronal density and microvasculature volume fraction across sites, which our analysis corroborates when restricted to the cerebral cortical GM alone (Table 6). Importantly, while this variation of capillary density with neuronal density is significant across cortical sites, it is by no means large enough to maintain a constant E/N ratio across cerebral cortical sites within the mouse brain: even within the cerebral cortical GM, the E/N ratio still decreases with increasing neuronal density (Figures 7A,B and Table 6), which is indicative of less energy availability per neuron in these sites. Therefore, it remains true for the cerebral cortex alone that our data suggest that energy availability per neuron is supply-limited.

There are presently no direct data available on rates of energy use per neuron, whether in different structures or species, to test the hypothesis that larger neurons use more energy (whether due to supply or demand); all measurements so far have been of energy use per gram of tissue (Mink et al., 1981; Karbowski, 2007), not per neuron. A necessary first step to establishing whether larger neurons *use* more energy, be it due to increased demand, supply, or both, is comparing average energy use per neuron where neurons have different sizes, both within and across species. The practical impediment here is that measuring energy use requires bringing live animals to the lab. We propose that local capillary density, which can be measured readily and efficiently in 2D images of thin brain sections, is a good proxy and viable alternative to estimating energy availability per volume as well as per neuron in fixed brain tissue. What makes it a good proxy is that local capillary density in fixed brain tissue correlates very well, and linearly, with resting blood flow and local rate of glucose use in both rat (Klein et al., 1986; Borowsky and Collins, 1989) and macaque brains (Noda et al., 2002). A direct validation of the correspondence between local capillary density and resting metabolic rate is reported in the accompanying study (Ventura-Antunes et al., 2022), in which we find that the small variations in the local vascular fraction measured as in the present study across sites in the rat brain are a very good approximation of measurements of local energy cost at rest in each structure. We thus expect that systematic analyses of local capillary densities will provide a powerful tool to circumvent the limitations to studying energy use per neuron in living animals, and open the way to comparative studies of energy availability per neuron in brains of species that are unlikely ever to be brought alive to laboratory settings.

It is possible that blood flow rates through capillaries vary across sites depending on factors such as network geometry and branch order, and also vary over time, according to variations in blood pressure in arterioles. However, red blood cell transit times in the brain have been shown to be relatively uniform across resting awake and stimulated conditions, with rates of oxygen transfer determined primarily by capillary length density (Jesperson and Ostergaard, 2012). Most importantly, the demonstration that local rates of blood flow in the awake brain are strongly, and linearly, correlated with both capillary density and rates of glucose use (Klein et al., 1986; Borowsky and Collins, 1989) provides the necessary evidence that local capillary density is a useful proxy for local rates of energy supply and use at rest.

While we examine fixed tissue and can make no claims about physiological properties and dynamic changes in neurovascular coupling (Leithner and Royl, 2014), the relatively homogeneous distribution of the capillary bed in the perfused tissue compared to highly variable neuronal densities, in the presence of constant overall blood flow in the brain (Mintun et al., 2001; Sato et al., 2011; Smith and Ainslie, 2017), does inform that, per neuronal cell body, there is more energy available in those sites with lower neuronal densities (with few and larger neurons; Mota and Herculano-Houzel, 2014) than in sites of high neuronal density. These findings are compatible with a scenario where neurons compete for limited energy supply, rather than the usually presumed scenario in which larger neurons and/or neurons with more synapses *demand* more energy, which is then provided as the capillary bed adapts to those demands during development. A limited energy supply constrained by the density of the capillary bed would have direct consequences for the level of neuronal and synaptic activity that can be sustained across brain sites, in line with our previous suggestion that fundamental aspects of neuronal structure and function are constrained by energy supply across species (Herculano-Houzel, 2011). It remains to be determined whether these constraints manifest themselves during development, for instance through the self-regulation of the numbers of synapses that can form and remain active, or simply through self-regulation of levels of activity (as in synaptic homeostasis; Turrigiano, 2012).

The present findings have fundamental implications for brain health and normal and diseased aging. First, they suggest that the heightened vulnerability of the human WM to ischemia (Wang et al., 2016) may be primarily due to its low capillary density compared to GM structures, and not simply to its large relative volume. Second, as cortical locations with high neuron densities have far less blood supplied per neuron than locations with low neuronal densities, individual neurons in “crowded” cortical areas are likely to be much more vulnerable to aging and pathologies that compromise circulation and/or metabolic capacity. Most remarkably, the hippocampus, whose granular layer contains most of its neurons and exhibits some of the lowest values of E/N found here, is one of the brain structures most highly vulnerable to hypoxia (Hossmann, 1999). Finally, some of the regions first and most vulnerable to Alzheimer’s disease are cortical areas with high resting metabolism (Buckner et al., 2005). While that might be indicative of higher capillary densities that would increase energy availability as a whole, it is possible that this is offset by very high neuronal densities, such as those found in the entorhinal cortex and hippocampus here, leading to low values of E/N and thus particularly high vulnerability to hypoxic insults. We are currently investigating the effects of aging on capillary density in the brain, and the possibility that local variation in E/N correlates with brain tissue vulnerability in normal and diseased aging.

## Data availability statement

The raw data supporting the conclusions of this article will be made available by the authors, without undue reservation.

## Ethics statement

All animal use in this project was approved by the Committee on Ethical Animal Use of the Health Sciences Center (CEUA-CCS), Federal University of Rio de Janeiro (UFRJ), with protocol number 01200.001568/2013-87.

## Author contributions

SH-H conceived the study. LV-A executed all data acquisition. Both authors analyzed the data and wrote the manuscript.

## References

[B1] Ames I. I. I. A. (2000). CNS energy metabolism as related to function. *Brain Res. Rev.* 34 42–68. 10.1016/S0165-0173(00)00038-2 11086186

[B2] Aschoff J. Günther B. Kramer K. (1971). “Energiehaushalt und Thermoregulation,” in *Physiologie des Menschen*, eds Gauer O. Kramer K. Jung R.. (Berlin:München-Wien).

[B3] Attwell D. Laughlin S. B. (2001). An energy budget for signaling in the grey matter of the brain. *J. Cereb. Blood Flow Metab.* 21 1133–1145. 10.1097/00004647-200110000-00001 11598490

[B4] Banavar J.-R. Damuth J. Maritan A. Rinaldo A. (2002). Supply–demand balance and metabolic scaling. *Proc. Natl. Acad. Sci. U.S.A.* 99 10506–10509. 10.1073/pnas.162216899 12149461 PMC124956

[B5] Boero J. A. Ascher J. Arregui A. Rovainen C. Woolsey T. A. (1999). Increased brain capillaries in chronic hypoxia. *J. Appl. Physiol.* 86 1211–1219. 10.1152/jappl.1999.86.4.1211 10194205

[B6] Borowsky I. W. Collins R. C. (1989). Metabolic anatomy of brain: A comparison of regional capillary density, glucose metabolism, and enzyme activities. *J. Comp. Neurol.* 288 401–413. 10.1002/cne.902880304 2551935

[B7] Braitenberg V. Schüz A. (eds). (1998). “Cortical architectonics,” in *Cortex: Statistics and Geometry of Neuronal Connectivity.* (Berlin: Springer), 135–137. 10.1007/978-3-662-03733-1_27

[B8] Buckner R. L. Snyder A. Z. Shannon B. J. LaRossa G. Sachs R. Fotenos A. F. (2005). Molecular, structural, and functional characterization of Alzheimer’s disease: Evidence for a relationship between default activity, amyloid, and memory. *J. Neurosci.* 25 7709–7717. 10.1523/JNEUROSCI.2177-05.2005 16120771 PMC6725245

[B9] Franklin K. B. J. Paxinos G. (1997). *The Mouse Brain in Stereotaxic Coordinates.* San Diego,CA: Academic Press.

[B10] Gundersen H. J. G. Jensen E. B. V. Kiêu K. Nielsen J. J. J. O. M. (1999). The efficiency of systematic sampling in stereology—reconsidered. *J. Microsc.* 193 199–211. 10.1046/j.1365-2818.1999.00457.x 10348656

[B11] Hainsworth F. R. Collins B.-G. Wolf L. L. (1977). The function of torpor in hummingbirds. *Physiol. Zool.* 50 215–222. 10.1086/physzool.50.3.30155724

[B12] Harris J. Jolivet R. Attwell D. (2012). Synaptic energy use and supply. *Neuron* 75 762–777. 10.1016/j.neuron.2012.08.019 22958818

[B13] Herculano-Houzel S. (2011). Scaling of brain metabolism with a fixed energy budget per neuron: Implications for neuronal activity, plasticity and evolution. *PLoS One* 6 e17514. 10.1371/journal.pone.0017514 21390261 PMC3046985

[B14] Herculano-Houzel S. Manger P. R. Kaas J. H. (2014). Brain scaling in mammalian evolution as a consequence of concerted and mosaic changes in numbers of neurons and average neuronal cell size. *Front. Neuroanat.* 8:77. 10.3389/fnana.2014.00077 25157220 PMC4127475

[B15] Herculano-Houzel S. Rothman D. L. (2022). From a demand-based to a supply-limited framework of brain metabolism. *Front. Integr. Neurosci.* 16:818685. 10.3389/fnint.2022.818685 35431822 PMC9012138

[B16] Herculano-Houzel S. Watson C. R. Paxinos G. (2013). Distribution of neurons in functional areas of the mouse cerebral cortex reveals quantitatively different cortical zones. *Front. Neuroanat.* 7:35. 10.3389/fnana.2013.00035 24155697 PMC3800983

[B17] Hossmann K. A. (1999). “The hypoxic brain,” in *Hypoxia*, Roach R. C. Wagner P. D. Hackett P. H. (eds) (Boston, MA: Springer), 155–169. 10.1007/978-1-4615-4711-2_14

[B18] Howarth C. Peppiatt-Wildman C. M. Attwell D. (2010). The energy use associated with neural computation in the cerebellum. *J. Cereb. Blood Flow Metab.* 30 403–414. 10.1038/jcbfm.2009.231 19888288 PMC2859342

[B19] Hudetz A. G. (1997). Blood flow in the cerebral capillary network: A review emphasizing observations with intravital microscopy. *Microcirculation* 4 233–252. 10.3109/10739689709146787 9219216

[B20] Jesperson S. N. Ostergaard L. (2012). The roles of cerebral blood flow, capillary transit time heterogeneity, and oxygen tension in brain oxygenation and metabolism. *J. Cereb. Blood Flow Metab.* 32 264–277. 10.1038/jcbfm.2011.153 22044867 PMC3272609

[B21] Karbowski J. (2007). Global and regional brain metabolic scaling and its functional consequences. *BMC Biol.* 5:18. 10.1186/1741-7007-5-18 17488526 PMC1884139

[B22] Klein B. W. Schrock K. H. Vetterlein F. (1986). Interdependency of local capillary density, blood flow, and metabolism in rat brains. *Am. J. Physiol. Heart Circul. Physiol.* 251 H1333–H1340. 10.1152/ajpheart.1986.251.6.H1333 3098116

[B23] Kuschinsky W. Paulson O. B. (1992). Capillary circulation in the brain. *Cerebrovasc. Brain Metab. Rev.* 4 261–286. 1389958

[B24] Lauwers F. Cassot F. Lauwers-Cances V. Puwanarajah P. Duvernoy H. (2008). Morphometry of the human cerebral cortex microcirculation: General characteristics and space-related profiles. *Neuroimage* 39 936–948. 10.1016/j.neuroimage.2007.09.024 17997329

[B25] Leach R. M. Treacher D. F. (1998). Oxygen transport. Tissue hypoxia. *Br. Med. J.* 317 1370–1373. 10.1136/bmj.317.7169.1370 9812940 PMC1114253

[B26] Leithner C. Royl G. (2014). The oxygen paradox of neurovascular coupling. *J. Cereb. Blood Flow Metab.* 34 19–29. 10.1038/jcbfm.2013.181 24149931 PMC3887356

[B27] Lin A. L. Fox P. T. Hardies J. Duong T. Q. Gao J.-H. (2010). Nonlinear coupling between cerebral blood flow, oxygen consumption, and ATP production in human visual cortex. *Proc. Natl. Acad. Sci. U.S.A.* 107 8446–8451. 10.1073/pnas.0909711107 20404151 PMC2889577

[B28] Madsen P. L. Holm S. Herning M. Lassen N. A. (1993). Average blood flow and oxygen uptake in the human brain during resting wakefulness: A critical appraisal of the Kety—Schmidt technique. *J. Cereb. Blood Flow Metab.* 13 646–655. 10.1038/jcbfm.1993.83 8314918

[B29] Magistretti P. J. (2006). Neuron–glia metabolic coupling and plasticity. *J. Exp. Biol.* 209 2304–2311. 10.1242/jeb.02208 16731806

[B30] Mink J. W. Blumenschine R. J. Adams D. B. (1981). Ratio of central nervous system to body metabolism in vertebrates: Its constancy and functional basis. *Am. J. Physiol. Reg. Integr. Comp. Physiol.* 241 R203–R212. 10.1152/ajpregu.1981.241.3.R203 7282965

[B31] Mintun M. A. Lundstrom B. N. Snyder A. Z. Vlassenko A. G. Shulman G. K. Raichle M. E. (2001). Blood flow and oxygen delivery to human brain during functional activity: Theoretical modeling and experimental data. *Proc. Natl. Acad. Sci. U.S.A.* 98 6859–6864. 10.1073/pnas.111164398 11381119 PMC34443

[B32] Mota B. Herculano-Houzel S. (2014). All brains are made of this: A fundamental building block of brain matter with matching neuronal and glial masses. *Front. Neuroanat.* 8:127. 10.3389/fnana.2014.00127 25429260 PMC4228857

[B33] Mullen R. J. Buck C. R. Smith A. M. (1992). NeuN, a neuronal specific nuclear protein in vertebrates. *Development* 116 201–211. 10.1242/dev.116.1.201 1483388

[B34] Noda A. Ohba H. Kakiuchi T. Futatsubashi M. Tsukada H. Nishimura S. (2002). Age-related changes in cerebral blood flow and glucose metabolism in conscious rhesus monkeys. *Brain Res.* 936 76–81. 10.1016/S0006-8993(02)02558-1 11988232

[B35] Pawlik G. Rackl A. Bing R. J. (1981). Quantitative capillary topography and blood flow in the cerebral cortex of cats: An in vivo microscopic study. *Brain Res.* 208 35–58. 10.1016/0006-8993(81)90619-3 7470927

[B36] Pellerin L. Magistretti P.-J. (2004). Neuroenergetics: Calling upon astrocytes to satisfy hungry neurons. *Neuroscientist* 10 53–62. 10.1177/1073858403260159 14987448

[B37] Rolfe D. F. Guy C. B. (1997). Cellular energy utilization and molecular origin of standard metabolic rate in mammals. *Physiol. Rev.* 77 731–758. 10.1152/physrev.1997.77.3.731 9234964

[B38] Sato K. Ogoh S. Hirawawa A. Oue A. Sadamoto T. (2011). The distribution of blood flow in the carotid and vertebral arteries during dynamic exercise in humans. *J. Physiol.* 589 2847–2856. 10.1113/jphysiol.2010.204461 21486813 PMC3112559

[B39] Schölvinck M. L. Howarth C. Attwell D. (2008). The cortical energy needed for conscious perception. *Neuroimage* 40 1460–1468. 10.1016/j.neuroimage.2008.01.032 18321731 PMC2330065

[B40] Schüz A. Palm G. (1989). Density of neurons and synapses in the cerebral cortex of the mouse. *J. Comp. Neurol.* 286 442–455. 10.1002/cne.902860404 2778101

[B41] Smith K. J. Ainslie P. N. (2017). Regulation of cerebral blood flow and metabolism during exercise. *Exp. Physiol.* 102 1356–1371. 10.1113/EP086249 28786150

[B42] Tsai P. S. Kaufhold P. Blinder P. Friedman B. Drew P. J. Karten H. J. (2009). Correlations of neuronal and microvascular densities in murine cortex revealed by direct counting and colocalization of nuclei and vessels. *J. Neurosci.* 29 14553–14570. 10.1523/JNEUROSCI.3287-09.2009 19923289 PMC4972024

[B43] Turrigiano G. (2012). Homeostatic synaptic plasticity: Local and global mechanisms for stabilizing neuronal function. *Cold Spring Harb. Perspect. Biol.* 4:a005736. 10.1101/cshperspect.a005736 22086977 PMC3249629

[B44] Ventura-Antunes L. Dasgupta O. M. Herculano-Houzel S. (2022). Resting rates of blood flow and glucose use per neuron are Proportional to number of endothelial cells available per neuron across sites in the rat brain. *Front. Integr. Neurosci.* 16:821850. 10.3389/fnint.2022.821850 35757100 PMC9226568

[B45] Wang Y. Liu G. Hong D. Chen F. Ji X. Cao G. (2016). White matter injury in ischemic stroke. *Progr. Neurobiol.* 141 45–60. 10.1016/j.pneurobio.2016.04.005 27090751 PMC5677601

[B46] Zheng J. H. A. Coggan A. R. Zhang X. Bashir A. Muccigrosso D. Peterson L. R. (2014). Noncontrast skeletal muscle oximetry. *Magn. Res. Med.* 71 318–325. 10.1002/mrm.24669 23424006 PMC3661680

[B47] Zhu F. Cizeron M. Qiu Z. Benavides-Piccione R. Kopanitsa M. V. Skene N. G. (2018). Architecture of the mouse brain synaptome. *Neuron* 99 781–799. 10.1016/j.neuron.2018.07.007 30078578 PMC6117470

